# *Flavobacterium columnare* ferric iron uptake systems are required for virulence

**DOI:** 10.3389/fcimb.2022.1029833

**Published:** 2022-10-17

**Authors:** Rachel A. Conrad, Jason P. Evenhuis, Ryan S. Lipscomb, David Pérez-Pascual, Rebecca J. Stevick, Clayton Birkett, Jean-Marc Ghigo, Mark J. McBride

**Affiliations:** ^1^ Department of Biological Sciences, University of Wisconsin-Milwaukee, Milwaukee, WI, United States; ^2^ National Center for Cool and Cold Water Aquaculture, Agricultural Research Service, United States Department of Agriculture (USDA), Kearneysville, WV, United States; ^3^ Institut Pasteur, Université de Paris-Cité, CNRS UMR 6047, Genetics of Biofilms Laboratory, Paris, France

**Keywords:** *Flavobacterium columnare*, virulence, iron acquisition, outer membrane siderophore receptor, heme binding protein

## Abstract

*Flavobacterium columnare*, which causes columnaris disease, is one of the costliest pathogens in the freshwater fish-farming industry. The virulence mechanisms of *F. columnare* are not well understood and current methods to control columnaris outbreaks are inadequate. Iron is an essential nutrient needed for metabolic processes and is often required for bacterial virulence. *F. columnare* produces siderophores that bind ferric iron for transport into the cell. The genes needed for siderophore production have been identified, but other components involved in *F. columnare* iron uptake have not been studied in detail. We identified the genes encoding the predicted secreted heme-binding protein HmuY, the outer membrane iron receptors FhuA, FhuE, and FecA, and components of an ATP binding cassette (ABC) transporter predicted to transport ferric iron across the cytoplasmic membrane. Deletion mutants were constructed and examined for growth defects under iron-limited conditions and for virulence against zebrafish and rainbow trout. Mutants with deletions in genes encoding outer membrane receptors, and ABC transporter components exhibited growth defects under iron-limited conditions. Mutants lacking multiple outer membrane receptors, the ABC transporter, or HmuY retained virulence against zebrafish and rainbow trout mirroring that exhibited by the wild type. Some mutants predicted to be deficient in multiple steps of iron uptake exhibited decreased virulence. Survivors of exposure to such mutants were partially protected against later infection by wild-type *F. columnare.*

## Introduction

*Flavobacterium columnare* causes columnaris disease in cultured fish including rainbow trout, channel catfish, tilapia, and many others (Bullock et al., 1986; Wagner et al., 2006; Declercq et al., 2013). Columnaris outbreaks are a chronic issue for the global aquaculture industry leading to significant economic losses and setbacks in the advancement of sustainable fish farming. Current control methods rely heavily on antibiotics, which can lead to the spread of antibiotic resistance. A better understanding of *F. columnare* pathogenesis is needed to develop alternate methods, such as efficient vaccines to prevent outbreaks. *F. columnare* pathogenesis mechanisms are still poorly understood despite recent studies examining potential virulence factors such as the type IX secretion system (T9SS), chondroitin sulfate lyases, peptidases, cytolysins and siderophores (Li et al., 2015; Li et al., 2017; Conrad et al., 2022; Thunes et al., 2022).

Iron is required for growth and pathogenesis in most disease-causing bacteria (Ratledge and Dover, 2000). Although iron is abundant in nature, in the presence of O_2_ it is found primarily in the ferric form, which is sparingly soluble and thus difficult to utilize (Cartron et al., 2006; Krewulak and Vogel, 2008). In animals, most ferric iron is sequestered into host proteins such as transferrin, lactoferrin, and heme. The resulting low levels of free iron limit bacterial growth and infection (Krewulak and Vogel, 2008; Skaar, 2010).

Iron acquisition has been linked to virulence in many pathogens, including the fish pathogens *Edwardsiella ictaluri* (Abdelhamed et al., 2016), *Vibrio anguillarum* (Wolf and Crosa, 1986), *Aeromonas salmonicida* (Hirst et al., 1991), and *Flavobacterium psychrophilum* (Zhu et al., 2022). Several studies explored iron acquisition by *F. columnare* and related fish pathogens (Avendano-Herrera et al., 2005; Moller et al., 2005; Guan et al., 2013; Bruce et al., 2021). Genes predicted to be involved in iron uptake have been identified by analyses of *F. columnare* genomes (Tekedar et al., 2017; Zhang et al., 2017). Iron deprivation is known to alter expression of some of these genes and to alter virulence (Beck et al., 2016).

**Figure 1:**
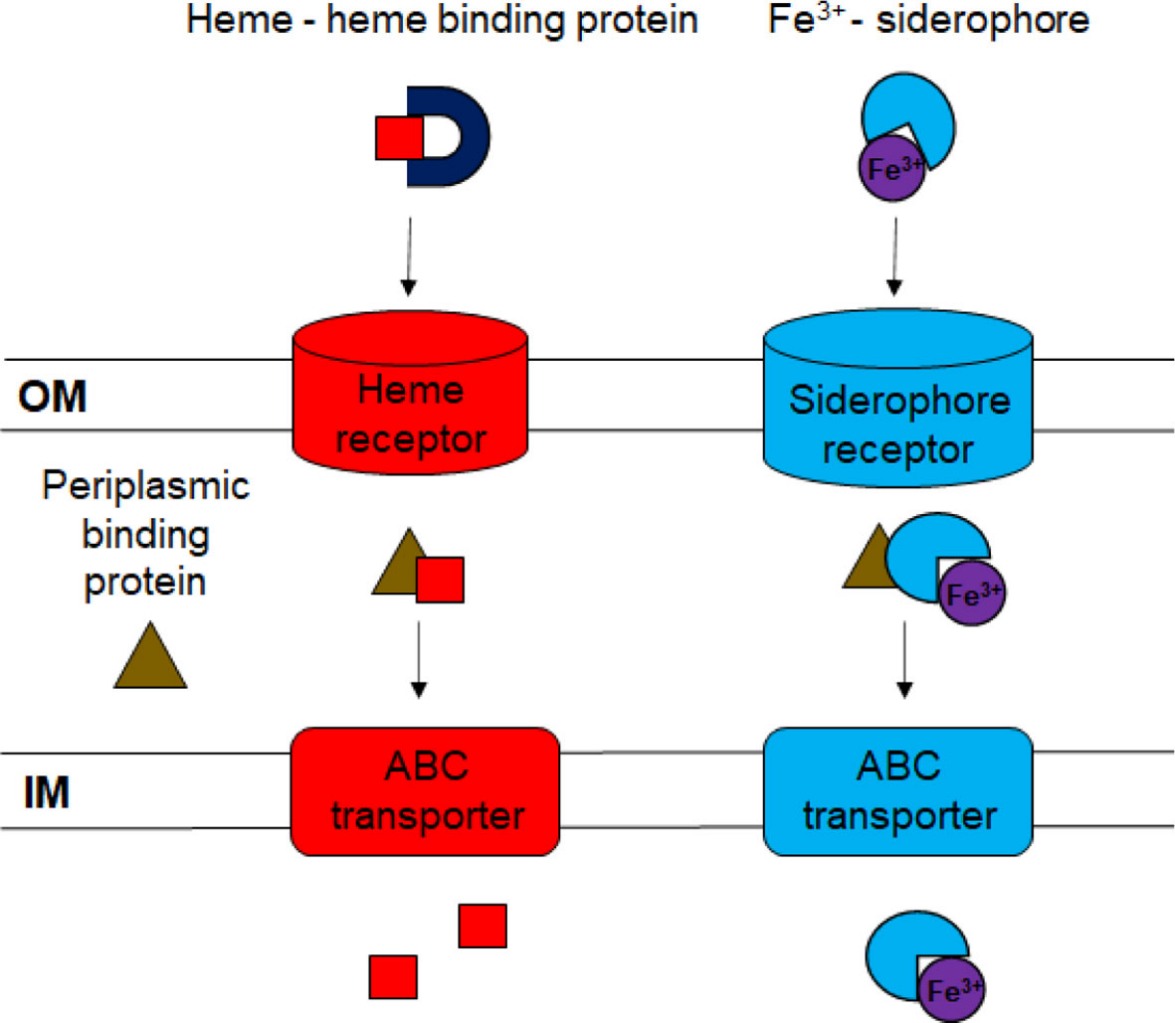
Diagram of ferric iron uptake systems. Ferric iron bound to siderophores or to heme is transported across the outer membrane (OM) using specific outer membrane receptors. The dark blue “U” represents the heme-binding protein HmuY. Periplasmic binding proteins deliver the ferri-siderophores and heme to ABC transporters in the inner membrane (IM), where they are transported to the cytoplasm.

Siderophores, small high-affinity ferric iron-chelators, are common components of iron acquisition systems used to compete with the host sequestration mechanisms (**Figure 1**). Siderophore-iron complexes (ferri-siderophores) are taken up by specific receptors on the bacterial cell surface (Sheldon et al., 2016). *F. columnare* produces siderophores under iron deprivation (Guan et al., 2013) and the expression of genes predicted to be involved in siderophore synthesis increases following exposure to fish mucus (Lange et al., 2018). *F. columnare* genes needed for siderophore production and secretion were recently identified and mutated (Conrad et al., 2022). Mutants deficient in siderophore production exhibited growth defects in iron limiting conditions but retained virulence against zebrafish and rainbow trout.

Heme is an iron source for some Gram-negative bacterial pathogens (Skaar, 2010). These bacteria use either of two mechanisms to acquire heme. The first involves binding heme directly using an outer membrane receptor whereas the second involves a soluble secreted protein that binds heme and interacts with an outer membrane receptor (Ratledge and Dover, 2000; Wandersman and Delepelaire, 2004). Heme acquisition is essential for growth and virulence of *Porphyromonas gingivalis*, a distant relative of *F. columnare* within the *Bacteroidota* phylum (Grenier et al., 2001; Smalley et al., 2011). Deletion of the *P. gingivalis* gene encoding the heme-binding protein HmuY led to reduced growth in an environment where serum was the only heme source (Olczak et al., 2015). In a more closely related member of the phylum, fish pathogen *F. psychrophilum*, a heme acquisition system that involves a HmuY-like protein, HfpY, for heme uptake was recently identified. Deletion of *hfpY* resulted in decreased virulence in rainbow trout fry (Zhu et al., 2022). Heme utilization genes and proteins have also been identified in other fish pathogenic bacteria, including members of the genera *Vibrio*, *Aeromonas*, and *Tenacibaculum* (Avendano-Herrera et al., 2005; Najimi et al., 2008; Ruiz et al., 2016). *F. columnare* produces HmuY, which is secreted by the T9SS and may facilitate heme uptake (Thunes et al., 2022).

Specific TonB-dependent outer membrane receptors transport ferri-siderophores and heme into the periplasm (Benson and Rivera, 2013; Sheldon et al., 2016). The cytoplasmic membrane protein TonB interacts with the outer membrane receptors and with ExbB and ExbD, which provide the energy for transport (Ratledge and Dover, 2000). ExbD has been demonstrated to be important for virulence of *F. psychrophilum* (Alvarez et al., 2008). Multiple TonB-dependent receptors have been described for ferric iron uptake, and expression of genes encoding these proteins is often upregulated in low-iron conditions (Braun, 2005; Bruce et al., 2021). Previous studies identified the *F. columnare* genes encoding the predicted TonB-dependent outer membrane siderophore receptors FhuA, IutA, and C6N29_04165 (Guan et al., 2013; Conrad et al., 2022), but their roles in iron utilization and virulence have not been explored further. The fate of ferri-siderophores and heme molecules that reach the periplasm has been studied in other Gram-negative bacteria (Davidson and Chen, 2004; Benson and Rivera, 2013; Sheldon et al., 2016). In these bacteria, specific periplasmic proteins bind siderophores and heme and deliver them to ATP-powered ABC (ATP-binding-cassette) transporters for transport across the cytoplasmic membrane (**Figure 1**).

In many bacteria, iron uptake systems are transcriptionally regulated by the Fur (ferric uptake regulator) protein. When intracellular iron levels are low, genes that encode outer membrane receptors for siderophores and heme are upregulated. When iron levels are high, Fur represses expression of these genes, limiting the toxic effects of excess iron (Carpenter et al., 2009). Deletion of *fur* results in decreased virulence in some bacteria (Wang et al., 2008; Santander et al., 2012; Ebanks et al., 2013). A *fur* mutant of the fish pathogen *Edwardsiella ictaluri* was attenuated for virulence in zebrafish and channel catfish, and fish exposed to the *fur* mutant by immersion were protected against later infection with wild-type cells (Santander et al., 2012).

In this study, we identified and deleted *F. columnare* genes predicted to be involved in ferric iron uptake. The mutants were used to demonstrate the importance of ferric acquisition systems for *F. columnare* growth in iron-limited conditions, and for ability to cause disease in zebrafish and in rainbow trout.

## Results

### Identification of *F. columnare* strain MS-FC-4 iron utilization genes

*F. columnare* genes involved in siderophore synthesis were recently identified and examined (Conrad et al., 2022). Bioinformatic analysis of the *F. columnare* MS-FC-4 genome identified additional genes predicted to be involved in ferric iron uptake (**Table 1**). Proteins encoded by these genes include the extracellular heme binding protein HmuY, 14 outer membrane receptors predicted to be involved in ferric iron uptake, the components of an ABC transporter for iron uptake across the cytoplasmic membrane, and proteins related to the ferric uptake regulator (Fur).

**Table 1:** Genes encoding proteins predicted to function in *F. columnare* strain MS-FC-4 ferric iron acquisition^a^.

Locus tag	Protein name	NCBI definition	Conserved domains^b^	Predicted function^c^	Protein localization^d^
**Heme uptake**					
C6N29_01035	HmuY	Hypothetical protein	HmuY_like super family (cl12009, pfam14064), Por_Secre_tail super family (cl40962, TIGR04183, pfam18962),	Heme binding	Outer Membrane/Extracellular
**TonB-dependent outer membrane siderophore receptors (TIGR01783)**					
C6N29_04165	FhuE	TonB-dependent siderophore receptor	OM channels super family (cl21487), TonB-dependent siderophore receptor (COG1629, TIGR01783,pfam00593, pfam07715)	Siderophore uptake	Outer Membrane
C6N29_09970	IutA^e^	TonB-dependent siderophore receptor	OM channels super family (cl21487), TonB-dependent siderophore receptor (TIGR01783, pfam00593, pfam07715)	Siderophore uptake	Outer Membrane
C6N29_10625	FhuA	TonB-dependent receptor	Ligand_gated_channel (cd01347), TonB-dependent siderophore receptor (TIGR01783, pfam00593, pfam07715), Outer membrane receptor for ferric coprogen (COG4773)	Siderophore uptake	Outer Membrane
**Other potential TonB-dependent outer membrane iron receptors**					
C6N29_00590		TonB-dependent receptor	OM channels super family (cl21487), Fe transport outer membrane receptor (COG1629), TonB-dependent receptor (pfam00593, pfam07715), CarboxypepD_reg-like domain (pfam13715)	Iron uptake	Outer Membrane
C6N29_01515		TonB-dependent receptor	OM channels super family (cl21487), Fe transport outer membrane receptor (COG1629), TonB-dependent receptor (pfam00593, pfam07715)	Iron uptake	Outer Membrane
C6N29_02560		TonB-dependent receptor	OM channels super family (cl21487), Fe transport outer membrane receptor (COG1629, TonB-dependent receptor (pfam00593, pfam07715)	Iron uptake	Outer Membrane
C6N29_02745		TonB-dependent receptor	OM channels super family (cl21487), Fe transport outer membrane receptor (COG1629), TonB dependent receptor (pfam00593, pfam07715)	Iron uptake	Outer Membrane
C6N29_06865		TonB-dependent receptor	OM_channels super family (cl21487), Outer membrane receptor for ferrienterochelin and colicins (COG4771), TonB-dependent receptor (pfam00593, pfam07715)	Iron uptake	Outer Membrane
C6N29_07090		TonB-dependent receptor	OM channels super family (cl21487), Fe transport outer membrane receptor (COG1629), TonB dependent receptor (pfam00593, pfam07715)	Iron uptake	Outer Membrane
C6N29_09245		TonB-dependent receptor	OM channels super family (cl21487), Outer membrane receptor for ferrienterochelin and colicins (COG4771), TonB-dependent receptor (pfam07715)	Iron uptake	Outer Membrane
C6N29_11000		TonB-dependent receptor	OM_channels super family (cl21487), Outer membrane receptor for ferrienterochelin and colicins (COG4771), TonB-dependent receptor (pfam00593, pfam07715), CarbopepD_reg_2 (pfam13715)	Iron uptake	Outer Membrane
C6N29_12875	FecA	TonB-dependent receptor	OM channels super family (cl21487), Outer membrane receptor for Fe^3+^-dicitrate (COG4772), TonB-dependent receptor (pfam00593)	Iron uptake	Outer Membrane
C6N29_12885		TonB-dependent receptor	OM channels super family (cl21487), Outer membrane receptor for ferrienterochelin and colicins (COG4771), TonB-dependent receptor (pfam07715)	Iron uptake	Outer Membrane
C6N29_13215		TonB-dependent receptor	OM channels super family (cl21487), Outer membrane receptor for ferrienterochelin and colicins (COG4771), TonB-dependent receptor (pfam00593, pfam07715)	Iron uptake	Outer Membrane
**Iron ABC transporter**					
C6N29_01845	ABC type iron transporter binding protein	ABC transporter substrate-binding protein	TroA-like super family (cl00262), inorganic ion transport (COG0614), periplasmic binding protein (pfam01497)	Iron uptake	Periplasmic
C6N29_01850	Iron transporter	Iron ABC transporter	FecCD transport family (pfam01032), inorganic ion transport (COG0609),	Iron uptake	Inner Membrane
C6N29_01855	ABC transporter ATPase	ABC transporter ATP-binding protein	FepC (COG1120), ABC transporter (pfam00005)	Iron uptake	Cytoplasmic
**Ferric iron uptake regulator**					
C6N29_04015		Transcriptional repressor	Fur-like (cd07153, COG0735, pfam01475)	Ferric uptake regulation	Cytoplasmic
C6N29_09035	Fur	Transcriptional repressor	Fur-like (cd07153, COG0735, pfam01475)	Ferric uptake regulation	Cytoplasmic

a Genes involved in siderophore synthesis and export within the *sidL* locus, and *iucX*, were excluded from this analysis because they were discussed in a previous study (Conrad et al., 2022).

b Conserved domains as assigned by NCBI and by the Joint Genome Institute Integrated Microbial Genomes & Microbiomes (IMG/M version 6.0 [https://img.jgi.doe.gov/m]) (Chen et al., 2021). TIGRFAM, pfam, smart, cl, cd, or COG numbers are indicated.

c Function predicted based on conserved domains and gene organization.

d Location of each protein was predicted using psortb 3.0 (Yu et al., 2010).

e IutA was previously identified in a previous study on *F. columnare* siderophore synthesis (Conrad et al., 2022).

The *F. columnare* T9SS-secreted HmuY protein exhibits weak similarity to *P. gingivalis* heme-binding protein HmuY and is predicted to aid in heme uptake in members of the *Bacteroidota* (Smalley et al., 2011; Zhu et al., 2022). The *F. columnare* HmuY protein contains a conserved HmuY super family domain (pfam14064) that is also seen in the *P. gingivalis* HmuY and *F. psychrophilum* HfpY proteins.

Outer membrane receptors transport siderophore-associated ferric iron, and other chelated forms of ferric iron into the cell. We identified genes encoding the predicted outer membrane receptors IutA, FhuA, FhuE, and FecA. *F. columnare* IutA, FhuA and FhuE each contain a conserved TonB-dependent siderophore receptor domain (TIGR01783). These proteins are predicted to transport siderophores, as they do in other bacteria (Andrews et al., 2003; Hider and Kong, 2010). *F. columnare* IutA was identified as part of the multi-gene siderophore biosynthesis locus (*sidL*) (Conrad et al., 2022). IutA is a well-studied outer membrane receptor in *Escherichia coli* that is involved in uptake of iron-bound siderophores (Williams, 1979; Carbonetti and Williams, 1984). The *F. columnare* IutA protein, (20% amino acid identity to *E. coli* IutA over 171 amino acids) may have a similar role. A previous study identified the *F. columnare* gene encoding the predicted TonB-dependent outer membrane siderophore receptor FhuA in *F. columnare* strain ATCC 23463 (Guan et al., 2013). The *F. columnare* strain MS-FC-4 FhuA protein is 99.9% identical to the previously identified *F. columnare* FhuA, 55% identical to *F. psychrophilum* FhuA, and 21.5% identical to *E. coli* FhuA. The *E. coli* FhuE receptor also transports siderophore bound iron across the outer membrane (Sauer et al., 1990). The predicted *F. columnare* FhuE protein is 21.1% identical to the *E. coli* protein and shares the conserved TonB-dependent siderophore receptor domain (TIGR01783). FecA transports siderophores and ferric citrate across the outer membrane in *E. coli* and other bacteria (Yue et al., 2003; Krewulak and Vogel, 2008). *F. columnare* FecA, which is 20% identical to *E. coli* FecA protein and 63.7% identical to the *F. psychrophilum* FecA, may function similarly. These FecA proteins all contain the conserved domain for ferric dicitrate outer membrane receptors (COG4772) that is characteristic of this protein. Other predicted outer membrane iron receptors were identified and are listed in **Table 1**. These diverse outer membrane receptors suggest that *F. columnare* can use ferric iron complexed with diverse organic molecules. Genes predicted to encode TonB (C6N29_03345), ExbB (C6N29_03360), and ExbD (C6N29_03350 and C6N29_03355) were also identified in *F. columnare* strain MS-FC-4. These proteins presumably form a complex that energizes transport of diverse molecules across the outer membrane *via* TonB-dependent outer membrane receptors, as they do in other bacteria (Ratliff et al., 2022).

ABC transporters are used to transport bound iron across the cytoplasmic membrane (Andrews et al., 2003). We identified genes encoding three components of a predicted ABC transporter involved in iron uptake. The periplasmic binding protein (C6N29_01845) and cytoplasmic membrane transporter protein (C6N29_01850) contain conserved domains predicted for ferric iron transport as indicated in **Table 1**. These are the only *F. columnare* ABC transporter proteins with identified conserved domains related specifically to iron uptake, and thus they may be important for ferric iron acquisition.

The bacterial ferric uptake regulator (Fur) is a global regulator that controls expression of many iron acquisition genes involved in siderophore synthesis, iron transport, and iron storage (Carpenter et al., 2009). The *F. columnare* strain ATCC 23463 Fur protein was previously identified and is similar to Fur proteins of other bacteria (Guan et al., 2013). Two *F. columnare* strain MS-FC-4 genes predicted to encode Fur-like proteins were identified. C6N29_09035 encodes Fur that is 100% identical to the previously studied *F. columnare* ATCC 23463 Fur and exhibits 98.7% or greater identity to Fur proteins from six other *F. columnare* strains with complete or nearly complete genome sequences. The other gene related to *fur*, C6N29_04015, is not found in most *F. columnare* strains. It is part of an approximately 37 kbp region of the *F. columnare* MS-FC-4 genome that is not present in most *F. columnare* strains, and that may be of foreign origin (Conrad et al., 2022). The protein encoded by C6N29_04015 is 27.9% identical to *F. columnare* Fur (C6N29_09035).

### Deletion of genes predicted to be involved in ferric iron utilization

To determine the functions of the *F. columnare* genes described above, deletion mutants were constructed targeting different steps in the pathways used to transport different chelated forms of ferric iron. Mutants lacking genes encoding the predicted extracellular heme-binding protein HmuY, the outer membrane receptors FhuA, FhuE, and FecA, and the components of the predicted iron transporting cytoplasmic membrane ABC transporter were generated (**Table 2**). Mutants lacking multiple combinations of these genes were also constructed. In some cases the 14 kbp *sidL* locus, which spans *iutA* and other genes involved in siderophore synthesis, export, and uptake (Conrad et al., 2022) was also deleted. We also attempted, unsuccessfully, to delete C6N29_09035, which encodes the predicted Fur protein. Plasmid pRC41 was constructed to obtain the *fur* deletion, and this was transferred to *F. columnare* and integrated into the genome. However, the second recombination event, which was expected to result in approximately equal numbers of wild-type and *fur* deletion mutant colonies, only gave rise to strains that were wild type at the *fur* locus.

**Table 2:** Strains used in this study.

Strain	Description	Source or reference
***E. coli* strains**		
DH5αMCR	Strain used for general cloning	Life Technologies (Grand Island, NY)
S17-1 λ pir	Strain used for conjugation	(de Lorenzo and Timmis, 1994)
***F. columnare* strains**		
MS-FC-4	Wild type	(Evenhuis et al., 2016; Bartelme et al., 2018)
FCB89	Δ*hmuY*	This study
FCB108	Δiron ABC transporter (C6N29_01845, C6N29_01850, C6N29_01855)	This study
FCB113	Δ*fhuA*	This study
FCB115	Δ*sidL*, siderophore synthesis region (C6N29_09920, C6N29_09925, C6N29_09930, C6N29_09935, C6N29_09940, C6N29_09945, C6N29_09950, C6N29_09955, C6N29_09960, C6N29_09965, C6N29_09970)	(Conrad et al., 2022)
FCB140	Δ*sidL* ΔABC transporter	This study
FCB146	Δ*sidL* ΔABC transporter Δ*fhuA*	This study
FCB160	*ΔfhuE*	This study
FCB162	*ΔfhuA ΔfhuE*	This study
FCB165	*ΔfecA*	This study
FCB174	Δ*sidL* ΔABC transporter Δ*fhuA* Δ*iucX*	This study
FCB176	Δ*fhuA* Δ*fhuE* Δ*fecA*	This study
FCB195	Δ*sidL* ΔABC transporter Δ*fhuA* Δ*iucX* Δ*fhuE*	This study
FCB227	Δ*sidL* ΔABC transporter complemented by ABC transporter genes inserted into the chromosome	This study
FCB239	Δ*sidL* ΔABC transporter Δ*fhuA* Δ*iucX* complemented by ABC transporter genes inserted into the chromosome	This study
FCB240	Δ*sidL* ΔABC transporter Δ*fhuA* Δ*iucX* complemented by *iucX* inserted into the chromosome	This study
FCB241	Δ*sidL* ΔABC transporter Δ*fhuA* Δ*iucX* Δ*fhuE* complemented by *fhuE* inserted into the chromosome	This study
FCB248	Δ*fhuA* Δ*fhuE* Δ*fecA* complemented by *fhuE* inserted into the chromosome	This study

### Δ*hmuY* mutant did not exhibit growth defects in tryptone-salts medium with or without added yeast extract

**Figure 2:**
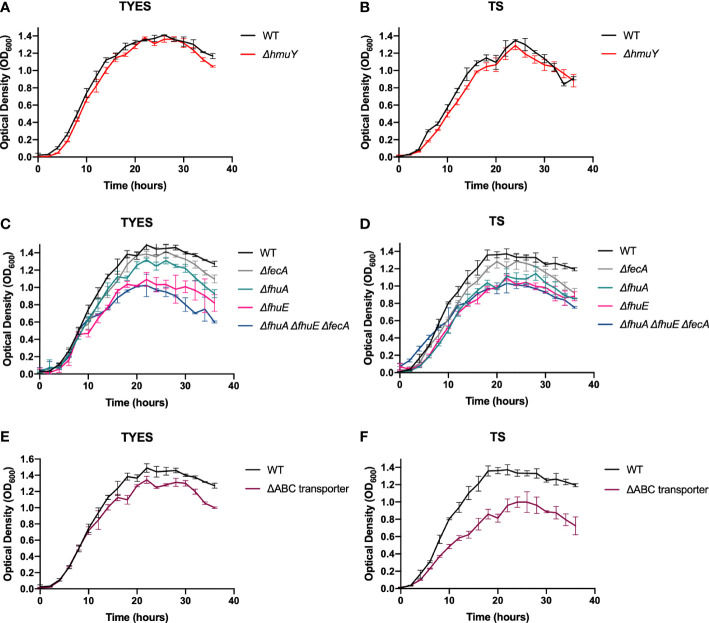
Growth of *F. columnare* wild type (WT) and iron mutant strains in iron-rich (TYES) and iron-limited (TS) medium. Δ*hmuY* mutant grown in TYES **(A)** and in TS **(B)**. *ΔfecA, ΔfhuA, ΔfhuE*, and *ΔfhuA ΔfhuE ΔfecA* mutants grown in TYES **(C)** and in TS **(D)**. Significant differences in peak cell density were seen in TYES between the WT and *ΔfhuA* (P < 0.05), WT and *ΔfhuE* (P < 0.0001), WT and *ΔfhuA ΔfhuE ΔfecA* (P < 0.0001). Significant differences in peak cell density were seen in TS between the WT and *ΔfhuA* (P < 0.001), WT and *ΔfhuE* (P < 0.0001), WT and *ΔfhuA ΔfhuE ΔfecA* (P < 0.0001). ΔABC transporter mutant grown in TYES **(E)** and in TS **(F)**. Significant differences in peak cell density were seen in TS between the WT and ΔABC transporter mutant (P < 0.0001). Wild type (WT) was included as a control in each experiment. Strains were grown at 28°C with shaking (200 rpm) and measurements were taken every two hours for 36 hours. Error bars represent standard error of the mean.

*F. columnare* wild type and iron acquisition mutants were grown in tryptone yeast extract salts (TYES) (Farmer, 2004), and in TS media, to examine for growth defects. TS medium is TYES medium without yeast extract. Yeast extract contains ferric iron and heme, and TS medium thus has lower levels of ferric iron. There is approximately 50 µg iron per g of yeast extract (Bridson and Brecker, 1970). TYES contains 0.4 g yeast extract per L, so we anticipate about 20 µg iron from yeast extract per L of TYES medium. The Δ*hmuY* mutant exhibited similar growth to wild type in both TYES and TS (**Figures 2A, B**), suggesting that HmuY had little effect on *F. columnare* growth under these conditions.

### Mutants lacking proteins that transport ferric iron across the outer membrane or the inner membrane exhibited growth defects in TS and TYES media

**Figure 3:**
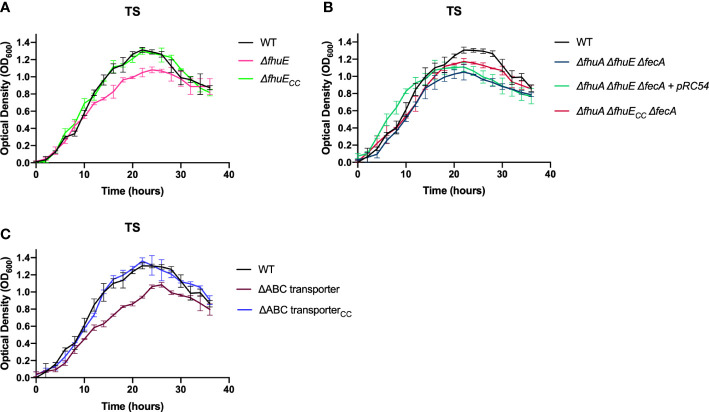
Growth of *F.columnare* wild type, outer membrane receptor deletion mutants, ABC transporter deletion mutant, and complemented mutants in iron-limited (TS) medium. **(A)** Wild type (WT), Δ*fhuE*, and Δ*fhuE* mutant complemented by inserting *fhuE* back into the chromosome. “CC” indicates chromosomal complementation. Significant differences in peak cell density were seen between the WT and Δ*fhuE* (P < 0.0001). **(B)** WT, Δ*fhuA* Δ*fhuE* Δ*fecA* mutant, Δ*fhuA* Δ*fhuE* Δ*fecA* mutant complemented with plasmid pRC54 (expresses *fhuE*), and Δ*fhuA* Δ*fhuE* Δ*fecA* mutant complemented by inserting *fhuE* back into the chromosome. Significant differences in peak cell density were seen between the WT and Δ*fhuA* Δ*fhuE* Δ*fecA* (P < 0.0001), WT and both complemented strains (P < 0.05), and Δ*fhuA* Δ*fhuE* Δ*fecA* and both complemented strains (P < 0.05). **(C)** WT, ΔABC transporter mutant, ΔABC transporter mutant complemented with plasmid pRC47 (expresses the ABC iron transporter genes), and ΔABC transporter mutant complemented by inserting the ABC transporter genes back into the chromosome. Significant differences in peak cell density were seen between the WT and ΔABC transporter mutant (P < 0.0001). Strains were grown at 28°C with shaking (200 rpm) and measurements were taken every two hours for 36 hours. Error bars represent standard error of the mean.

Mutants lacking single predicted outer membrane ferric iron receptors were examined for growth in TYES and TS (**Figures 2C, D**). The Δ*fecA* mutant grew similar to wild type. In contrast, the Δ*fhuA* mutant displayed a growth defect in TS and the Δ*fhuE* mutant exhibited growth defects in TS and in TYES. Chromosomal complementation of Δ*fhuE* restored growth (**Figure 3A**). The triple outer membrane receptor mutant *ΔfhuA ΔfhuE ΔfecA* also exhibited growth defects in both TYES and TS media (**Figures 2C, D**). Complementation of the Δ*fhuA* Δ*fhuE* Δ*fecA* triple mutant with pRC54, which carries *fhuE*, or by chromosomal complementation with *fhuE* partially restored growth in TS (**Figure 3B**). This may suggest partial redundancy of function between the outer membrane receptors or indicate that they transport different chelated forms of iron.

The ABC transporter comprised of C6N29_01845, C6N29_01850, and C6N29_01855 is predicted to transport ferric iron across the inner membrane. We constructed a deletion spanning the three genes encoding the components of this transporter. The resulting ΔABC-transporter mutant exhibited greater growth defects in TS than in the more iron-rich TYES medium (**Figures 2E, F**), suggesting that ABC transporter-mediated iron transport is more important when iron is scarce. The mutant was complemented by inserting the ABC transporter genes, expressed from their native promoter, in their original position on the chromosome of the mutant. This strain, referred to as ΔABC transporter_CC_ (where ‘_CC_’ indicates chromosomal complementation by integration) grew similar to the wild type (**Figure 3C**).

### Mutants deficient in multiple steps of iron transport exhibited growth defects in TYES and in TS

**Figure 4:**
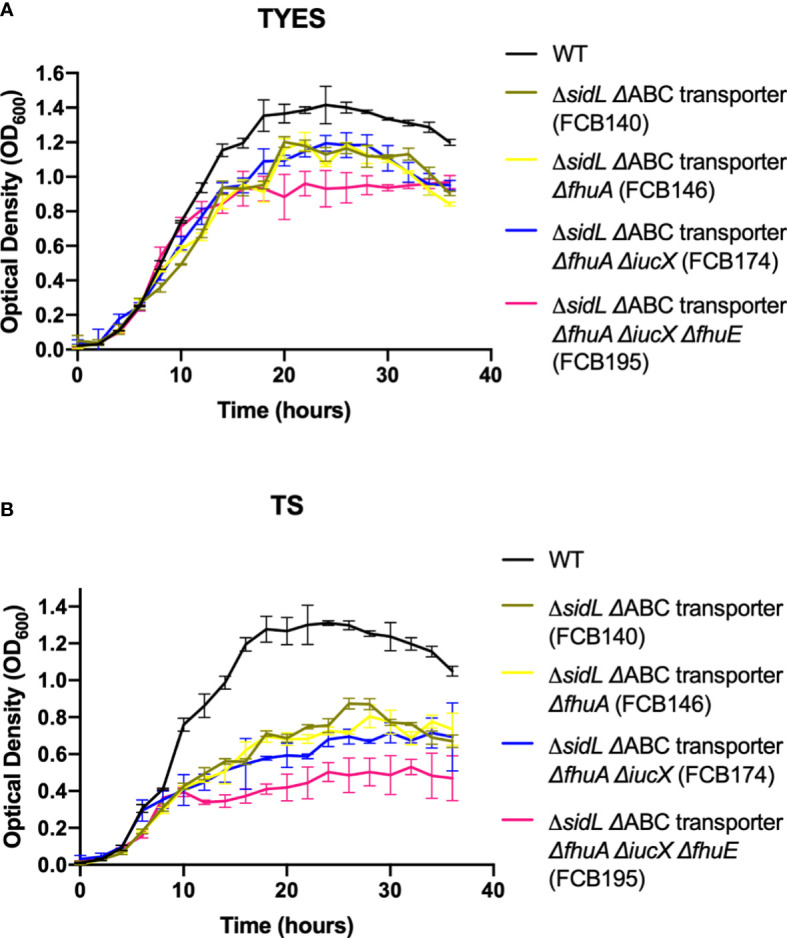
Growth of *F. columnare* wild type and mutants predicted to be deficient in multiple steps of iron uptake in iron-rich (TYES) and iron-limited (TS) medium. **(A)** Growth of wild type (WT), Δ*sidL* ΔABC transporter mutant (FCB140), Δ*sidL* ΔABC transporter Δ*fhuA* mutant (FCB146), Δ*sidL* ΔABC transporter Δ*fhuA* Δ*iucX* mutant (FCB174), and Δ*sidL* ΔABC transporter Δ*fhuA* Δ*iucX* Δ*fhuE* (FCB195) mutants in TYES. *sidL* is a roughly 14 kbp region containing genes involved in siderophore production, export, and regulation that was previously studied (Conrad et al., 2022). Significant differences in peak cell density were seen between the WT and FCB140, WT and FCB146, WT and FCB174 (P < 0.05), and WT and FCB195 (P < 0.0001). **(B)** Growth of the same strains described in panel ‘A’ in TS. Significant differences in peak cell density were seen between the WT and FCB140, WT and FCB146, WT and FCB174, and WT and FCB195 (P < 0.0001). Strains were grown at 28°C with shaking (200 rpm) and measurements were taken every two hours for 36 hours. Error bars represent standard error of the mean.

**Figure 5:**
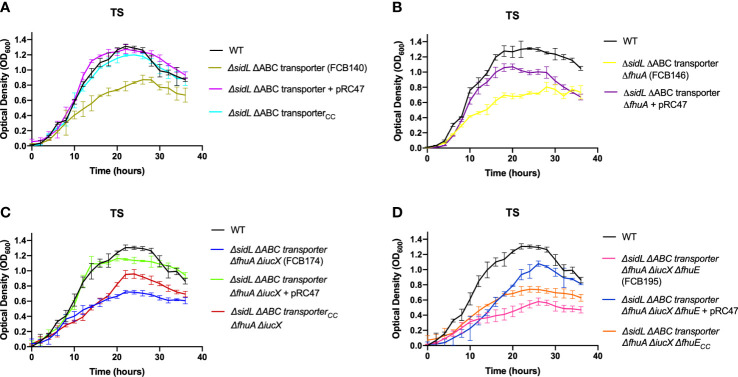
Growth of *F. columnare* wild type, mutants predicted to be deficient in multiple steps of iron uptake, and complemented mutants in iron-limited (TS) medium. **(A)** Wild type (WT), Δ*sidL* ΔABC transporter mutant (FCB140), FCB140 complemented with pRC47 (expresses the iron ABC transporter genes), and FCB140 complemented by inserting the ABC transporter genes back into the chromosome (“_CC_” indicates the gene(s) inserted into the chromosome). *sidL* is a roughly 14 kbp region containing genes involved in siderophore synthesis, export, and regulation that was previously studied (Conrad et al., 2022). Significant differences in peak cell density were seen between the WT and FCB140 (P < 0.0001). **(B)** Wild type, Δ*sidL* ΔABC transporter Δ*fhuA* mutant (FCB146), and FCB146 complemented with pRC47. Significant differences in peak cell density were seen between the WT and FCB146 (P < 0.0001), WT and complemented mutant (P < 0.01) and FCB146 and complemented mutant (P < 0.001). **(C)** Wild type, Δ*sidL* ΔABC transporter Δ*fhuA* Δ*iucX* mutant (FCB174), FCB174 complemented with pRC47, and FCB174 complemented by inserting the ABC transporter genes back into the chromosome. Significant differences in peak cell density were seen between the WT and FCB174 (P < 0.0001), WT and FCB174 complemented with pRC47 (P < 0.05), WT and FCB174 chromosomally complemented with the ABC transporter genes (P < 0.001), FCB174 and FCB174 complemented with pRC47 (P < 0.001), and FCB174 and FCB174 chromosomal complemented with the ABC transporter genes (P < 0.01). **(D)** Wild type (WT), Δ*sidL* ΔABC transporter Δ*fhuA* Δ*iucX* Δ*fhuE* mutant (FCB195), FCB195 complemented with pRC47, and FCB195 complemented by inserting *fhuE* back into the chromosome. Significant differences in peak cell density were seen between the WT and FCB195 (P < 0.0001), WT and FCB195 complemented with pRC47 (P < 0.05), WT and FCB195 chromosomally complemented with *fhuE* (P < 0.0001), and FCB195 and FCB195 complemented with pRC47 (P < 0.001). Strains were grown at 28°C with shaking (200 rpm) and measurements were taken every two hours for 36 hours. Error bars represent standard error of the mean.

Mutants carrying several mutations that reduce iron uptake in different ways demonstrated greater growth defects in both TS and TYES than did the single gene mutants (**Figure 4**). These mutants included those lacking siderophore production and lacking the iron ABC transporter (FCB140), and mutants lacking siderophore production, the iron ABC transporter, and one or more outer membrane receptors (FCB146, FCB174, FCB195). Mutants were complemented either using plasmids or by reinserting genes into the chromosome. Chromosomal complementation partially restored growth in TS for most of the mutants. Complementation of the multiple step iron mutants with pRC47, which encodes the components of the ABC transporter, fully restored growth in TS for the Δ*sidL* ΔABC transporter mutant and partially restored growth in TS for mutants where additional iron utilization genes were deleted (FCB146, FCB174, FCB195) (**Figure 5**). The most deficient mutant (FCB195), lacking genes for siderophore biosynthesis (*sidL* and *iucX*), multiple outer membrane receptors (*fhuA*, *fhuE*, and *iutA* [part of the *sidL* locus]), and the iron ABC transporter exhibited the greatest growth defect (**Figure 4**). The growth defects observed for the mutants described above could compromise their abilities to productively infect fish tissues, where competition for iron may be intense.

### Iron utilization mutants exhibited growth defects when available iron was decreased by chelation

Although TS medium has less iron than TYES medium, it may not be as restrictive as the more challenging conditions faced by bacteria growing in fish tissues, where proteins such as transferrin sequester iron. To mimic these conditions, deferiprone was added to TYES medium to bind free iron. In fish tissues, or in our *in vitro* experiments, *F. columnare* siderophores may compete with other chelators to obtain iron for bacterial growth (Conrad et al., 2022). We examined how disruption of other parts of the iron uptake pathway affect *F. columnare* growth in the presence of a chelator.

The minimum amount of deferiprone needed to prevent growth was determined for each strain (**Table 3**). The wild-type strain required the highest deferiprone concentration, 728 µM, to prevent growth. Supplementation with ferric chloride (FeCl_3_) restored growth, suggesting that the inhibitory effects of deferiprone were the result of iron chelation rather than some other toxic effect. Δ*hmuY*, Δ*fhuA*, Δ*fecA*, and ΔABC transporter mutants needed a similar concentration of chelator as the wild-type strain to prevent growth, suggesting that these genes are not individually critical for ferric iron uptake in chelated iron conditions. Less deferiprone was needed to prevent growth of the Δ*fhuE* mutant or the Δ*fhuA* Δ*fhuE* Δ*fecA* triple mutant, indicating that FhuE may be a more important outer membrane receptor for iron acquisition under these conditions. Previously, we showed that growth of siderophore gene deletion mutants was prevented by much lower concentrations of deferiprone (Conrad et al., 2022). Here, the Δ*sidL* mutant, which lacks the 14 kbp region containing 11 siderophore synthesis, export, and uptake genes, was used as the starting point to construct mutants deficient for siderophore production and for other steps in ferric iron uptake. The concentration of chelator to prevent growth (50 µM) of the *ΔsidL Δ*ABC transporter mutant was similar to that seen for the *ΔsidL* mutant. Mutants lacking siderophore production, the ABC transporter, and one or more outer membrane receptors required as little as 25 µM of deferiprone to prevent growth. These data highlight that eliminating multiple iron uptake genes greatly hinders *F. columnare* growth in conditions where iron is chelated.

**Table 3:** *F. columnare* growth in TYES with iron chelator, with and without supplemental iron.

Strain	Concentration of chelator (deferiprone) required to prevent growth^a,b^	Concentration of added iron (FeCl_3_) needed to restore turbid growth^b,c^
Wild type	728 µM	100 µM
Δ*hmuY*	634 µM	80 µM
Δ*fhuA*	634 µM	80 µM
Δ*fecA*	634 µM	100 µM
Δ*fhuE*	514 µM	70 µM
Δ*fhuA* Δ*fhuE* Δ*fecA*	514 µM	50 µM
ΔABC transporter	586 µM	80 µM
*ΔsidL*	50 µM	10 µM
Δ*sidL* ΔABC transporter	50 µM	10 µM
Δ*sidL* ΔABC transporter Δ*fhuA*	25 µM	10 µM
Δ*sidL* ΔABC transporter Δ*fhuA* Δ*iucX*	25 µM	20 µM
Δ*sidL* ΔABC transporter Δ*fhuA* Δ*iucX* Δ*fhuE*	25 µM	20 µM

a The amounts listed indicate the minimum concentration of deferiprone to prevent *F. columnare* growth.

b Tubes were incubated in a rotator for 24 h at 28°C.

c The minimum concentration of ferric chloride needed to restore turbid *F. columnare* growth in the presence of chelator.

### Iron acquisition systems are required for virulence in zebrafish and rainbow trout

**Figure 6:**
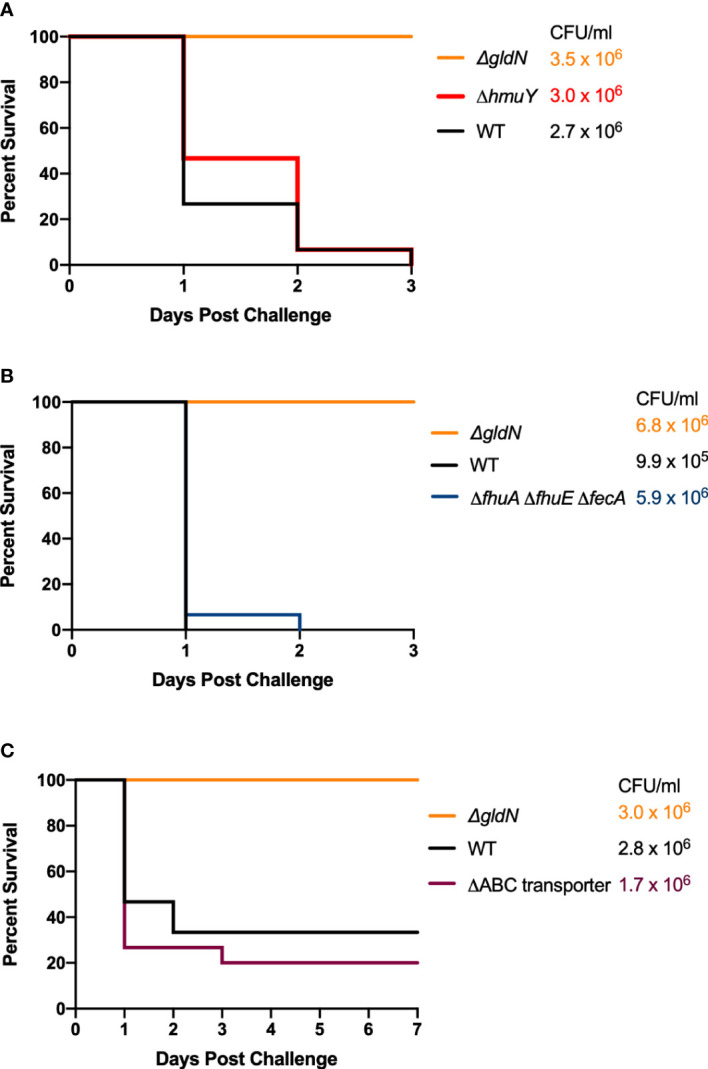
Virulence of *F. columnare* wild type and iron utilization mutants toward adult zebrafish. Zebrafish were exposed by immersion to *F. columnare* strains and percent survival was recorded. **(A)** Wild type (black), Δ*gldN* (orange), and Δ*hmuY* (red) mutants. The final challenge concentrations for each strain are shown in the corresponding color. **(B)** Wild type (black), Δ*gldN* (orange), and Δ*fhuA* Δ*fhuE* Δ*fecA* (blue), mutants. **(C)** Wild type (black), *ΔgldN* (orange), and ΔABC transporter (purple), mutants. The percent survival for fish challenged with the wild-type and Δ*hmuY*, Δ*fhuA* Δ*fhuE* Δ*fecA*, or ΔABC transporter mutant strains were not significantly different.

Wild-type *F. columnare* and iron utilization mutants grown in TYES medium were examined for their ability to kill zebrafish and rainbow trout at different stages in development. The secretion-deficient *F. columnare* T9SS mutant *ΔgldN*, which is avirulent, was used as a control in each of the challenges. Wild type *F. columnare* and the iron utilization mutants were examined for virulence against germ-free zebrafish larvae in a pilot challenge experiment (**Figure S1**). No significant differences in virulence were seen for any of the iron utilization mutants when compared to the wild type. When tested in adult zebrafish, mutants predicted to be deficient in heme binding (**Figure 6A**), outer membrane transport (**Figure 6B**), or inner membrane transport (**Figure 6C**), retained virulence similar to the wild type. Similar results were previously reported for siderophore deficient mutants (Conrad et al., 2022). TYES plates (containing tobramycin) streaked from the gills, fins, and skin of adult zebrafish that died following challenge displayed *F. columnare* colonies. These results demonstrate that the deletions examined that would affect single steps of the iron utilization pathway did not compromise virulence under the conditions used.

**Figure 7:**
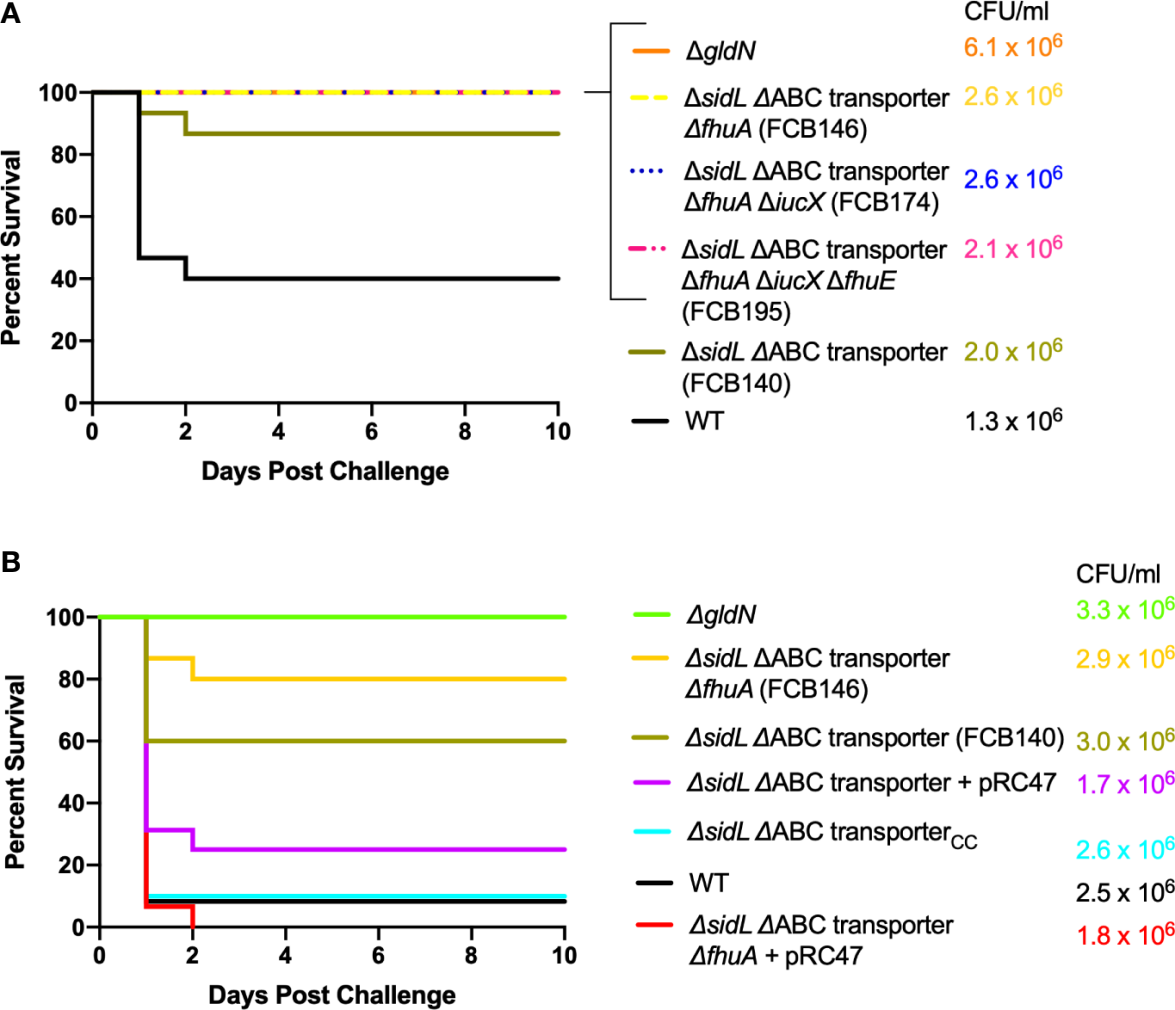
Virulence of *F. columnare* wild type and mutants predicted to be defective for multiple steps of iron uptake. Adult zebrafish were exposed by immersion to *F. columnare* strains and percent survival was recorded. **(A)** Wild type (black), Δ*gldN* (orange), *ΔsidL* ΔABC transporter (FCB140) (olive), Δ*sidL* ΔABC transporter Δ*fhuA* (FCB146) (yellow), *ΔsidL* ΔABC transporter *ΔfhuA ΔiucX* (FCB174) (blue), and Δ*sidL* ΔABC transporter *ΔfhuA ΔiucX ΔfhuE* (FCB195) (pink). The final challenge concentrations for each strain are shown in the corresponding color. Significant differences in the percent survival for fish challenged with *F. columnare* were seen between WT and FCB140 (P < 0.01), WT and FCB146 (P < 0.001), WT and FCB174 (P < 0.001), and WT and FCB195 (P < 0.001). **(B)** Wild type (black), Δ*gldN* (green), FCB140 (olive), FCB140 complemented with pRC47 (purple), FCB140 complemented by inserting the ABC transporter genes back into the chromosome (blue), FCB146 (yellow), and FCB146 complemented with pRC47 (red). pRC47 carries genes encoding the ABC transporter proteins. Significant differences in the percent survival for fish challenged with *F. columnare* were seen between WT and FCB140 (P < 0.01), WT and FCB146 (P < 0.0001), FCB140 and FCB140 complemented with pRC47 (P < 0.05), FCB140 and FCB140 chromosomally complemented with the ABC transporter genes (P < 0.01), and FCB146 and FCB146 complemented with pRC47 (P < 0.0001).

In contrast, mutants examined that should be deficient for iron uptake at multiple steps of the pathway were defective in virulence in adult zebrafish. The Δ*sidL* ΔABC transporter mutant exhibited decreased virulence and deletion mutant FCB146, which lacked *sidL*, the ABC transporter genes, and *fhuA*, failed to kill adult zebrafish (**Figure 7A**). Similar results were obtained for mutants in which *iucX* and *fhuE* were deleted from FCB146. Some zebrafish exposed to deletion mutants FCB140, FCB146, and FCB174 showed signs of columnaris disease including redness and lethargy during the ten-day study, while fish exposed to FCB195, which lacked the most iron utilization genes, displayed lethargy but not redness. These results suggest that the fish were infected by the mutants, but they recovered. Gills, fins, and skin of fish that died during the challenge experiment were examined for *F. columnare* cells by swabbing on TYES agar containing tobramycin. *F. columnare* colonies were observed in each case examined, suggesting that these fish, which had been exposed to wild type cells or to cells of the Δ*sidL* ΔABC transporter and Δ*sidL* ΔABC transporter Δ*fhuA* mutants, died from columnaris infections. Mutants were complemented either using plasmids or by reinserting the genes into the chromosome. Chromosomal complementation by insertion of the iron ABC transporter genes into FCB140, restored virulence and complementation with pRC47, which carries the same genes, into FCB140 and FCB146, also restored virulence (**Figure 7B**).

**Figure 8:**
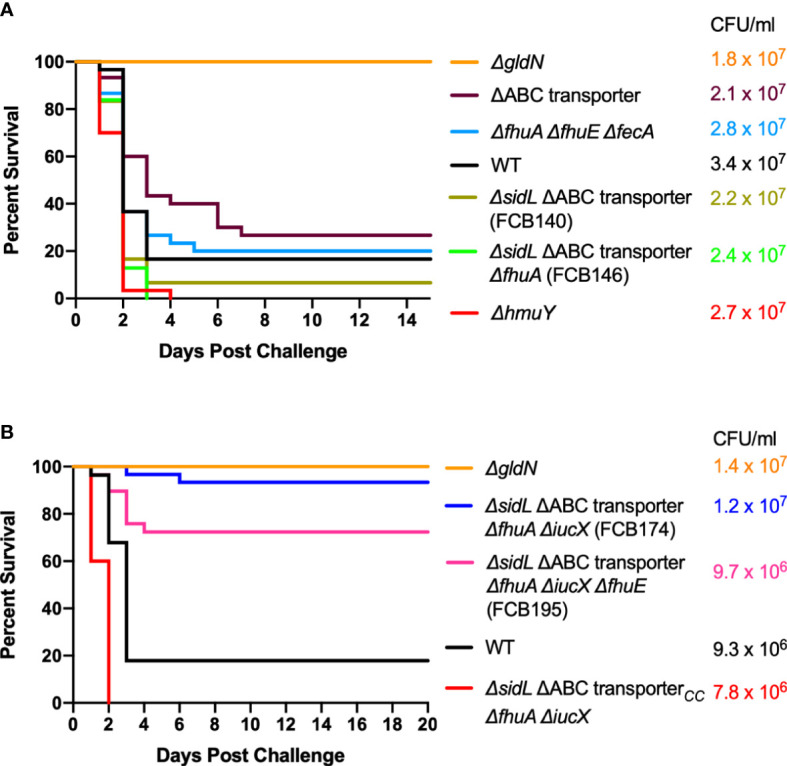
Virulence of *F. columnare* wild type and iron utilization mutants toward rainbow trout fry. **(A)** Wild type (black), *ΔgldN* (orange), *ΔhmuY* (red), *Δ*ABC transporter (purple), *ΔfhuA ΔfhuE ΔfecA* (blue), *ΔsidL* ΔABC transporter (olive), and Δ*sidL* ΔABC transporter Δ*fhuA* (green). The final challenge concentrations for each strain are shown in the corresponding color. **(B)** Wild type (black), *ΔgldN* (orange), *ΔsidL* ΔABC transporter *ΔfhuA ΔiucX* (FCB174) (blue), FCB174 chromosomally complemented with the ABC transporter genes (red), and Δ*sidL* ΔABC transporter *ΔfhuA ΔiucX ΔfhuE* (FCB195) (pink). Significant differences in the percent survival for fish challenged with *F. columnare* were seen between WT and FCB174 (P < 0.0001), WT and FCB195 (P < 0.0001), and FCB174 and FCB174 chromosomally complemented with the ABC transporter genes (P < 0.0001). The difference between FCB174 and FCB195 was not significant.

**Figure 9:**
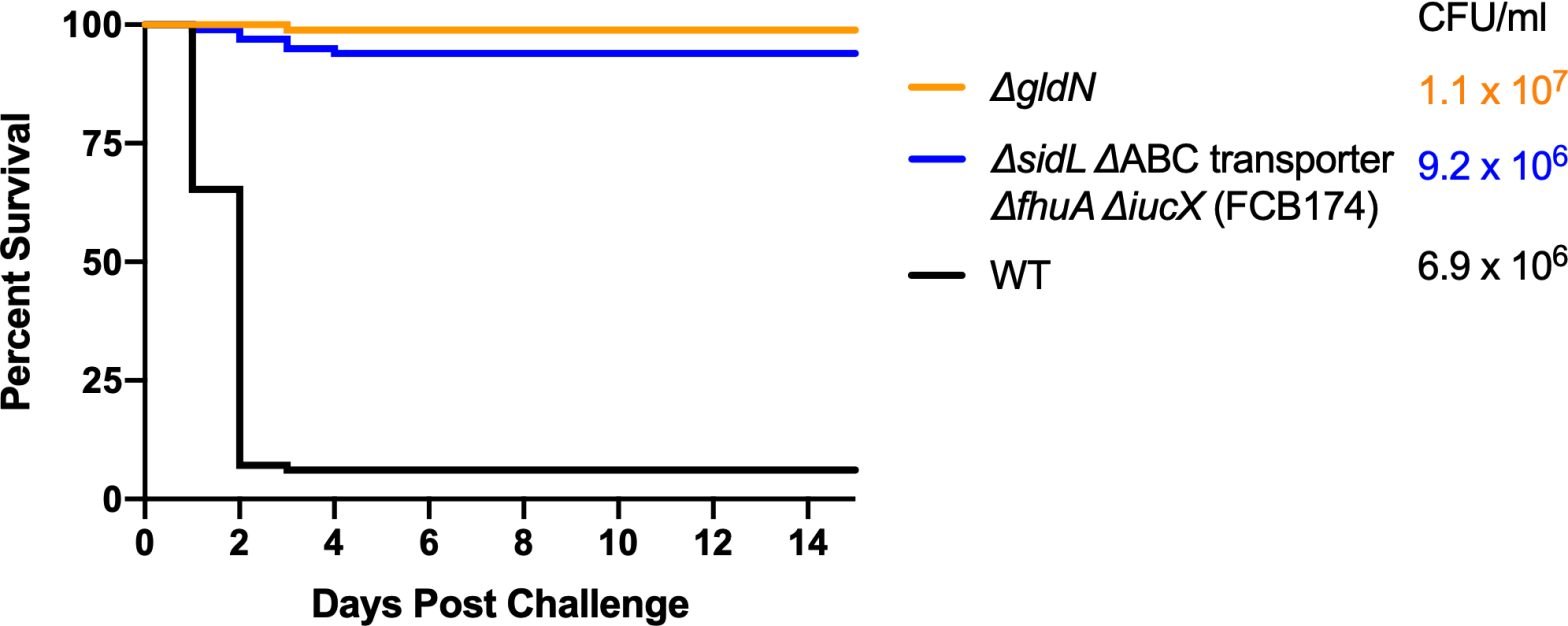
Virulence of *F. columnare* wild type and iron mutants toward rainbow trout alevin. Rainbow trout were exposed to the wild type (black), *ΔgldN* (orange), and *ΔsidL* ΔABC transporter *ΔfhuA ΔiucX* (FCB174) (blue) mutants. The final challenge concentrations for each strain are shown in the corresponding color. Significant differences in the percent survival for fish challenged were seen between the WT and FCB174 (P < 0.0001).

Wild-type *F. columnare* and mutants were also examined for their ability to kill rainbow trout juveniles (fry) and sac fry (alevin). The mutants predicted to be deficient in heme binding, outer membrane transport, or inner membrane transport retained virulence against fry similar to the wild type (**Figure 8A**). FCB174, lacking siderophore production, ABC transporter genes, and the outer membrane receptors FhuA and IutA exhibited the most dramatic virulence defect (**Figure 8B**). Similar virulence defects were seen for this mutant in rainbow trout alevin (**Figure 9**), demonstrating that this mutant was attenuated for virulence against rainbow trout at different developmental stages. Chromosomal complementation of this mutant with the ABC transporter genes restored virulence in rainbow trout fry (**Figure 8B**). *F. columnare* was reisolated from rainbow trout fry challenge mortalities, suggesting that the morbidity and mortality observed were due to *F. columnare* infections. 16S rRNA genes were amplified and analyzed as described in Methods. In each case the isolated bacteria belonged to genomovar 1 (genetic group 1), as expected for *F. columnare* strain MS-FC-4.

### Exposure to mutants predicted to be deficient in multiple steps of iron uptake provided partial protection against later challenge with the wild-type strain

**Figure 10:**
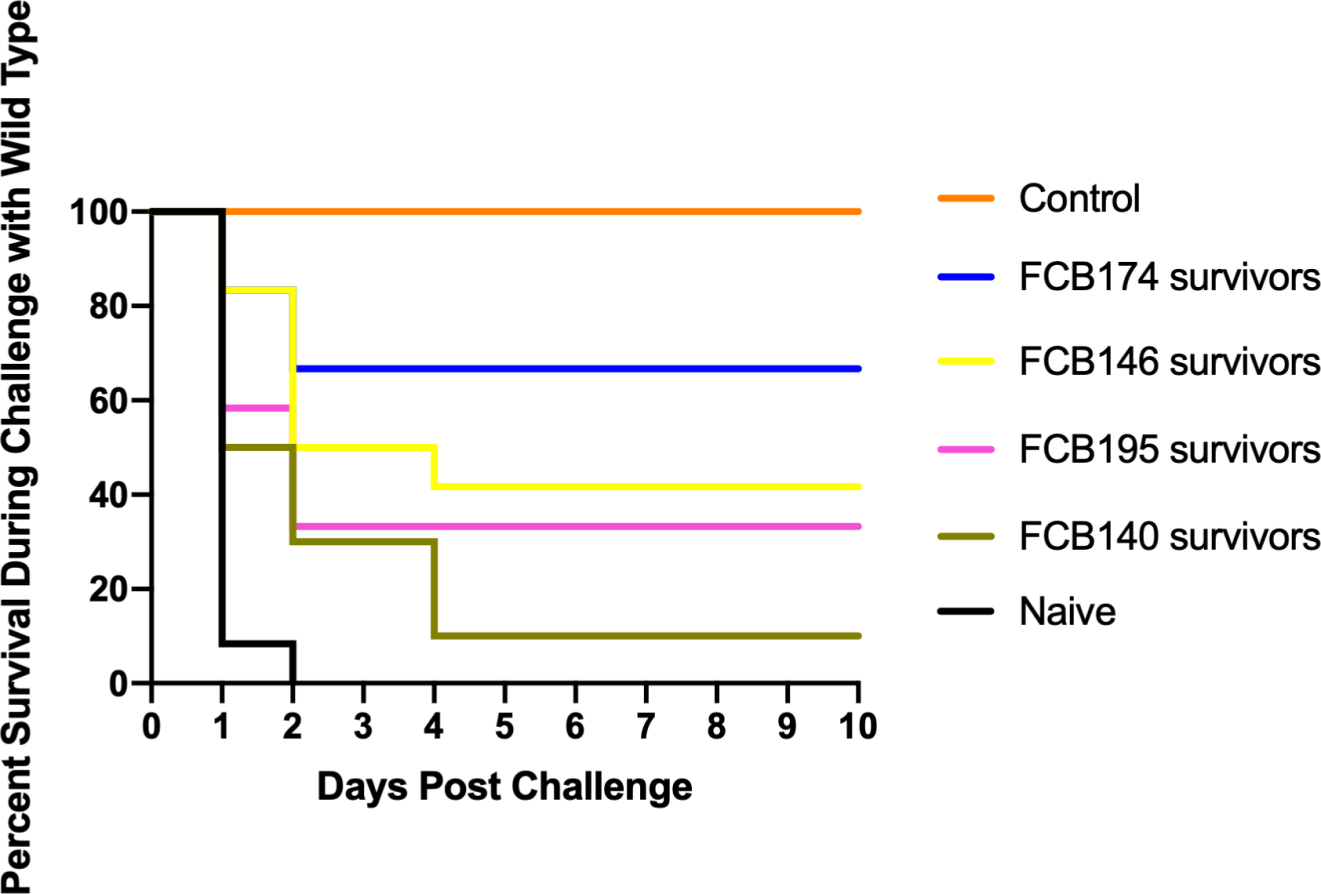
Challenge of zebrafish survivors with wild type *F. columnare*. Fish that survived exposure to mutants predicted to be deficient in multiple steps of iron uptake were examined for later resistance to challenge with wild-type cells. Naïve fish, FCB140 survivors, FCB146 survivors, FCB 174 survivors, and FCB195 survivors were maintained for 28 days and then exposed to *F. columnare* wild type (2.1 x 10^6^ CFU/ml). Naïve fish were not previously exposed to *F. columnare*. Control indicates fish exposed to Δ*gldN* mutant (6.3 x 10^6^ CFU/ml) instead of to wild type. Significant differences in the percent survival were seen for the naïve fish and FCB146 survivors (P < 0.001), naïve fish and FCB174 survivors (P < 0.0001), and naïve fish and FCB195 survivors (P < 0.01).

**Figure 11:**
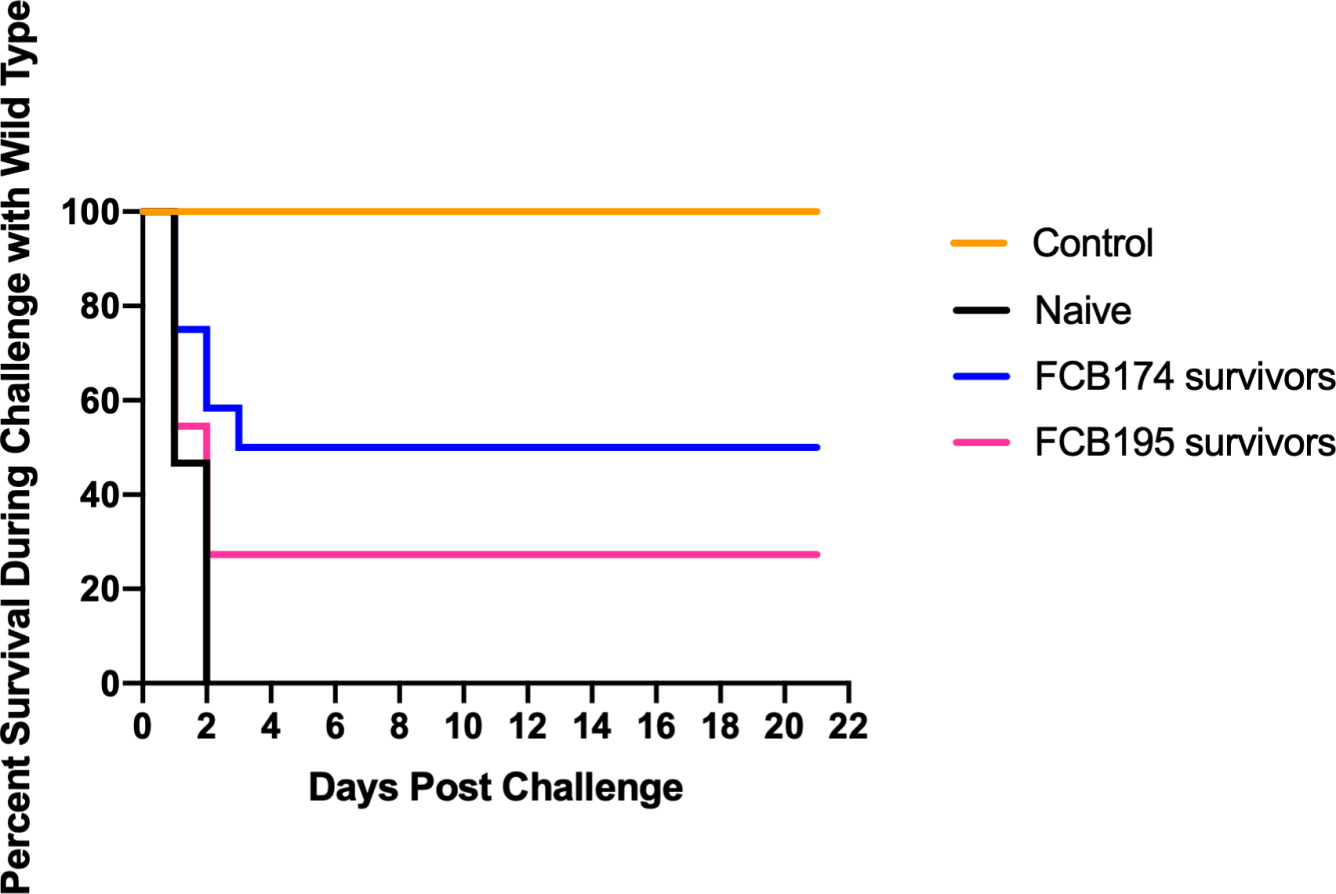
Challenge of rainbow trout fry survivors with wild type *F. columnare*. Fish that survived exposure to mutants predicted to be deficient in multiple steps of iron uptake were examined for later resistance to challenge with wild-type cells. Naïve fish, FCB 174 survivors, and FCB195 survivors were maintained for 28 days and then exposed to *F. columnare* wild type (7.1 x 10^6^ CFU/ml). Naïve fish were not previously exposed to *F. columnare*. Control indicates fish exposed to equivalent amount of TYES growth medium instead of to *F. columnare.* Significant differences in the percent survival were seen for the naïve fish and FCB174 survivors (P < 0.01).

A recent study demonstrated that rainbow trout that survived exposure to wild-type *F. columnar*e had elevated antibody levels, suggesting an adaptive immune response that might protect against future infection (Tongsri et al., 2020). *F. columnare* iron utilization mutants attenuated for virulence were examined for their ability to protect fish against later exposure to the wild type. Adult zebrafish and rainbow trout fry that survived exposure to attenuated mutants were later challenged with wild type *F. columnare*. Previous exposure to some of the multiple deletion mutants resulted in partial protection of zebrafish against later challenge with wild type *F. columnare* (**Figure 10**). When challenged with wild-type *F. columnare*, some of these fish displayed redness, lethargy, and rapid mouth and opercula movements, but many of these later recovered. Zebrafish initially challenged with mutant FCB174, which lacks siderophore production, the ABC transporter, and the outer membrane receptors IutA and FhuA, displayed the highest level of protection against later exposure to the wild type. Similar results were seen with rainbow trout that had survived exposure to FCB174 and were later challenged with wild type cells (**Figure 11**).

## Discussion

To gain a better understanding of *F. columnare* virulence factors, we identified genes for iron acquisition and analyzed mutants predicted to have defects in single or multiple steps in ferric uptake pathways. Mutants analyzed included those lacking the predicted extracellular heme-binding protein HmuY, multiple outer membrane receptors predicted to be involved in ferric iron uptake, and components of an ABC transporter predicted to transport iron across the cytoplasmic membrane.

Mutants with predicted defects in single or multiple steps in ferric uptake pathways were analyzed for growth defects in iron-rich and iron-limited conditions. Growth of the Δ*hmuY* mutant was similar to the wild type under iron-limited conditions, suggesting that heme acquisition did not contribute substantially to *F. columnare* growth under our *in vitro* conditions. HmuY may however play a role in iron acquisition in natural settings or during infection, as it does in *P. gingivalis* (Smalley et al., 2011; Olczak et al., 2015).

The outer membrane receptor mutant Δ*fhuE* and the Δ*fhuA* Δ*fhuE* Δ*fecA* triple outer membrane receptor mutant exhibited growth defects in both iron-rich and iron-limited media. Of the single receptors targeted, deletion of *fhuE* resulted in the greatest defect, suggesting that this receptor is important for iron acquisition under the conditions tested. A recent study in the pathogen *Acinetobacter baumannii* determined that FhuE was vital to iron acquisition, and when FhuE was bound to an inhibitor, cell growth was prevented (Tiwari et al., 2019). Further analysis of *A. baumannii* showed that under iron-limited conditions FhuE protein levels increased, and that it interacted with 32 different siderophores (Tiwari et al., 2019). More studies are needed to determine if *F. columnare* FhuE functions similarly.

The ΔABC transporter mutant exhibited growth defects in iron-limited conditions, but not in iron-rich conditions. This suggests that ABC transporter-mediated iron transport is more important when iron is scarce and that there are other mechanisms *F. columnare* uses to transport iron across the inner membrane. The Gram-negative pathogen *Pseudomonas aeruginosa* produces a ferric reductase enzyme that is localized to the periplasm (Schroder et al., 2003). This enzyme reduces ferric iron bound to the siderophore pyochelin to ferrous iron that is transported into the cell by a ferrous iron transport system (Cox, 1980; Schroder et al., 2003). It is unknown if *F. columnare* produces ferric reductases that function in this way, but *F. columnare* does have genes encoding proteins that are predicted to transport ferrous iron across the cytoplasmic membrane (Tekedar et al., 2017) (**Table S1**).

Growth defects seen in the mutants predicted to impact multiple steps in the iron uptake pathway were more severe than those for mutants predicted to affect an individual step in the pathway. An accumulation of mutations disrupting siderophore production, outer membrane transport, and inner membrane transport produced mutants that presumably had difficulty acquiring iron and thus did not grow well in low iron conditions.

*F. columnare* growth was further analyzed in conditions where iron was chelated, restricting bioavailability, to mimic possible conditions in fish tissue. Δ*hmuY*, Δ*fhuA*, Δ*fecA*, and ΔABC transporter mutants needed a similar concentration of chelator as the wild type to prevent growth. These strains produce siderophores, as observed on CAS plates (unpublished data), and they have iron acquisition machinery to transport the ferri-siderophores across the outer membrane, which could explain the high amount of chelator needed to prevent growth. The ΔABC transporter mutant likely has other mechanisms to transport iron across the inner membrane that have not been identified yet. Strains lacking *fhuE* were more sensitive to the chelator suggesting FhuE may be important for uptake of siderophores produced by *F. columnare*. FhuA, FecA, and the other identified outer membrane receptors may assist with the uptake of *F. columnare* siderophores or they could be used to take up siderophores produced by other bacteria as is seen in other pathogens (Magarinos et al., 1994; Sayyed et al., 2011). FhuA, FecA, and the 12 other predicted TonB-dependent iron receptors (**Table 1**) may take up iron complexed with other (non-siderophore) molecules. Many pathogens have outer membrane receptors for host iron sequestration proteins such as lactoferrin and transferrin (Krewulak and Vogel, 2008; Sheldon et al., 2016). Future studies are needed to determine the siderophores and other iron-carrying molecules that the *F. columnare* outer membrane receptors interact with. Mutants predicted to be deficient in multiple steps of iron uptake were inhibited by low concentrations of the iron chelator deferiprone, similar to the Δ*sidL* mutant (Conrad et al., 2022).

The ability to obtain iron is one of the key factors needed for successful infection of a host (Sheldon et al., 2016). However, *F. columnare* mutants that failed to produce siderophores remained virulent (Conrad et al., 2022). Similarly, here we showed that the loss of some individual iron acquisition functions did not seem to affect virulence. This may be the result of redundancies among the iron acquisition systems, as has been observed for other bacteria (Henderson and Payne, 1994; Mey et al., 2002; Dale et al., 2004; Williams et al., 2006). Some mutant strains compromised for three iron uptake steps (siderophore production, outer membrane transport, and inner membrane transport) were less virulent in adult zebrafish. These mutants also exhibited dramatic growth defects in iron-limited conditions. Perhaps their iron utilization defects prevented them from growing well enough in zebrafish to cause fatal infections. In contrast to the results with adult zebrafish, each of the mutants retained virulence against germ-free zebrafish larvae. There are several possible explanations for this difference. The immature immune systems of larval zebrafish may contribute to their increased sensitivity (Lam et al., 2004; Castro et al., 2015). The lack of a normal microbiota may also have made the larvae more sensitive to infection by *F. columnare*, as has previously been demonstrated (Stressmann et al., 2021).

Mutants predicted to be deficient in multiple steps of iron uptake also exhibited a significant reduction in virulence compared to the wild type, in rainbow trout. The Δ*sidL* ΔABC transporter Δ*fhuA* Δ*iucX* Δ*fhuE* mutant (FCB195) exhibited the greatest growth defects in our *in vitro* experiments, but it did not show the largest virulence defect in fish. One possible explanation for this could be a difference in gene expression. Expression of genes involved in iron uptake is often dependent on the amount of available iron present in the environment and in the cell (Andrews et al., 2003; Cartron et al., 2006). The above-mentioned mutant (FCB195) lacks genes for siderophore production, multiple outer membrane receptors, and an iron related ABC transporter, and may thus have very low intracellular iron levels even when external ferric iron is available. It is possible that low cellular iron resulted in the upregulation of iron acquisition genes or other virulence factors (Ochsner et al., 1995; Wang et al., 2008). This could have resulted in a higher mortality rate in rainbow trout even though the bacterium was more compromised for iron acquisition and growth than were other mutants that were less virulent.

Overall, the results observed for zebrafish and rainbow trout were similar, but not identical. The differences observed are not surprising given the many differences between these fish species and between the different maintenance and infection challenge conditions used in our studies. Zebrafish are warmwater fish, whereas rainbow trout require coldwater. The different temperatures may impact the bacterium and its virulence and could thus alter the outcome of infection (Declercq et al., 2013). We also challenged fish at different stages of development; larvae and adult for zebrafish, and alevin and fry for rainbow trout. The innate and adaptive immune responses that fish mount are affected by stage of development (Lam et al., 2004; Castro et al., 2015; Evenhuis et al., 2021). Finally, the zebrafish larvae were germ-free and thus more sensitive to *F. columnare* infection (Stressmann et al., 2021), whereas all other fish had complex microbiomes. This may explain why each of the iron uptake mutants examined retained the ability to kill germ-free zebrafish larvae, whereas some of the mutants exhibited reduced virulence toward the other fish examined.

Attenuated iron utilization mutants are potential vaccine candidates (Wang et al., 2019) that may protect against later infection by wild type *F. columnare*. Adult zebrafish survivors from exposure to mutants predicted to be deficient in multiple steps in iron uptake were later exposed to the wild-type strain and the fish were partially protected. Previous exposure to the Δ*sidL* ΔABC transporter Δ*fhuA* Δ*iucX* mutant provided the highest level of protection against later exposure to the wild-type strain. The mutant lacking the most iron uptake genes (Δ*sidL* ΔABC transporter Δ*fhuA* Δ*iucX* Δ*fhuE*) provided less protection. Anecdotally, adult zebrafish challenged with this mutant did not display redness like the fish exposed to the other multiple step deletion mutants. This may indicate that the mutant did not mount enough of an infection to generate a strong immune response. Rainbow trout survivors from an initial exposure to a mutant predicted to be deficient in multiple steps of iron uptake also exhibited partial protection against later challenge with the wild-type strain. Similar to the zebrafish results, the Δ*sidL* ΔABC transporter Δ*fhuA* Δ*iucX* mutant (FCB174) provided the highest level of protection. This mutant demonstrated decreased virulence in adult zebrafish and in multiple developmental stages (alevin and fry) of rainbow trout. Further studies may result in strains further attenuated for virulence that function as safe and protective vaccines.

## Methods

### Bacterial strains, plasmids, and growth conditions

*F. columnare* strain MS-FC-4 (Evenhuis and LaFrentz, 2016; Bartelme et al., 2018) was the wild-type strain used in this study and all mutants were derived from this strain. *F. columnare* strains were grown at 28°C to 30°C in tryptone yeast extract salts (TYES) medium (Holt et al., 1993; Cain and LaFrentz, 2007), which contains per L, 4 g tryptone, 0.4 g yeast extract, 0.5 g MgSO_4_·7H_2_O, and 0.5 g CaCl_2_·2H_2_O, pH adjusted to 7.2. *F. columnare* cultures used for rainbow trout challenges were grown in TYES-2xMg, which is identical to TYES except that it contains twice as much MgSO_4_. For some growth curves TS medium, which is TYES medium without yeast extract, was used. *E. coli* strains were grown at 37°C in lysogeny broth (LB) (Bertani, 1951). 100 µg/mL ampicillin was used to select for plasmids in *E. coli*. 5 µg/mL tetracycline (agar culture) or 2.5 µg/mL tetracycline (liquid cultures) were used to select for plasmids in *F. columnare*. 1 µg/mL tobramycin was used to counter-select against *E. coli* for conjugation experiments, and to eliminate most bacteria when isolating *F. columnare* from infected zebrafish. All *F. columnare* DNA fragments cloned in plasmids came from the wild-type strain MS-FC-4. The strains used in this study are listed in **Table 2**. Plasmids are listed in **Table S2**, and primers are listed in **Table S3**.

### Bioinformatic analysis

The *F. columnare* wild-type strain MS-FC-4 genome sequence (Bartelme et al., 2018), was examined for genes encoding proteins predicted to be involved in iron uptake. This was accomplished using the Joint Genome Institute’s Integrated Microbial Genomes and Microbiomes (IMG/M version 6.0 (Chen et al., 2021)) Function Profile Tool to search for sequences that encode proteins that have domains that belong to the families TIGR01783, TIGR04183, pfam00005, pfam00593, pfam01032, pfam01475, pfam01497, pfam07715, pfam14064, pfam18962, COG0609, COG0614, COG0735, COG1120, COG4771, COG4772 and COG4773. For TIGRFAMs, the trusted cutoffs assigned by The J. Craig Venter Institute (JCVI) that allow identification of the vast majority of family members with few false positives (Haft et al., 2013) were used. Proteins were also examined for other conserved domains using both the IMG/M (Chen et al., 2021) and the National Center for Biotechnology Information (NCBI) conserved domain searches (Marchler-Bauer and Bryant, 2004; Marchler-Bauer et al., 2011; Marchler-Bauer et al., 2015; Marchler-Bauer et al., 2017).

### Genetic manipulations and mutant construction

Plasmids were transferred from *E. coli* S17-1λpir into *F. columnare* by conjugation, and in-frame deletion mutants were constructed as previously described (Li et al., 2017; Conrad et al., 2022; Thunes et al., 2022). To delete *hmuY*, A 2.1 kbp region downstream of *hmuY* was amplified by PCR using Phusion DNA polymerase (New England Biolabs) and primers 2173 (adding a BamHI site) and 2174 (adding a SalI site). The product was digested with BamHI and SalI and ligated into pMS75 that had been digested with the same enzymes to produce pRC18. A 2.1 kbp region upstream of *hmuY* was amplified using primers 2171 (adding a KpnI site) and 2172 (adding a BamHI site). The product was digested with KpnI and BamHI and ligated into pRC18 that had been digested with KpnI and BamHI to generate pRC19. pRC19 was transferred to *F. columnare* MS-FC-4 by conjugation, and colonies with the plasmid integrated into the chromosome by recombination were obtained by selecting for tetracycline resistance. Resistant colonies were streaked for isolation on antibiotic plates and isolated colonies were grown in liquid without tetracycline to allow loss of the plasmid. The cells were plated on TYES media containing 5% sucrose and the mutant was obtained by selecting for sucrose resistance. PCR was performed to confirm the deletion. Other iron utilization genes were disrupted in a similar way, using the plasmids described in **Table S2**, and the primers listed in **Table S3**. Most deletion plasmids used the restriction enzyme pairs BamHI/SalI and KpnI/BamHI to digest the vector and inserts except for pRC42, pRC58, and pRC60. pRC42 used KpnI/BamHI and BamHI/SphI pairs. pRC58 used BamHI/SalI and SalI/SphI pairs. pRC60 used XmaI/BamHI and BamHI/SalI pairs.

### Plasmid complementation

A 3.1 kbp fragment spanning the ABC transporter genes was amplified using primers 2540 (adding a KpnI site) and 2541 (adding a PstI site). The product was digested with KpnI and PstI and ligated into the shuttle vector pCP23, which was digested with the same enzymes, to produce pRC47. The plasmid was transferred into the *F. columnare* ABC transporter mutant by conjugation and the resulting colonies were screened for tetracycline resistance. Resistant colonies were streaked for isolation on antibiotic containing plates and presence of the plasmid was confirmed by PCR. Complementation of other mutants was performed in a similar way, using the plasmids described in **Table S2**, and the primers listed in **Table S3**.

### Chromosomal complementation

A 6.9 kbp product spanning the ABC transporter genes and adjacent upstream and downstream regions was amplified using primers 2382A (adding a KpnI site) and 2385A (adding a SphI site). The product was digested with KpnI and SphI and ligated into pMS75 that had been digested with the same enzymes, to generate pRC68. The plasmid was transferred into the *F. columnare Δ*ABC transporter mutant by conjugation and colonies with the plasmid integrated into the chromosome by recombination were obtained by selecting for tetracycline resistance. Resistant colonies were streaked for isolation on agar plates containing tetracycline and isolated colonies were grown overnight in liquid without tetracycline to allow for a second recombination event, resulting in the loss of the plasmid from the chromosome. The cells were plated on TYES media containing 5% sucrose, selecting for sucrose resistance. The sucrose resistant colonies are either *Δ*ABC transporter mutants or complemented mutants where the ABC transporter genes had been inserted back into their original position in the chromosome. PCR was performed to confirm that the genes were reinserted. Chromosomal complementation of other mutants was performed similarly, using the plasmids described in **Table S2** and the primers listed in **Table S3**.

### *F. columnare* growth in iron-limited conditions

Stocks of *F. columnare* in TYES with glycerol added to 17.5%, stored at -80°C, were used to inoculate 20 mL of TYES broth and these were incubated for 14 hours at 28°C with shaking at 200 rpm. This was done to minimize cell clumping before inoculating the microtiter plate. Overnight cultures were standardized to an OD_600_ of 0.5, and 40 µL of culture was added to 960 µL of media (TYES or TS) per well in a 48 well microtiter plate. Cells were incubated in a CLARIOstar Microplate Reader (BMG Labtech, Ortenberg, Germany) at 28°C with shaking at 200 rpm. Readings were taken every 2 hours for 36 hours. Cultures were measured in triplicate in the microtiter plates and growth experiments were performed twice for each strain.

### *F. columnare* growth in chelated iron conditions

*F. columnare* strains were streaked from freezer stocks onto TYES agar and incubated at 30°C for 24 hours. Growth from TYES agar was then used to inoculate 5 mL of TYES broth, which was incubated overnight at 28°C with rotation. 15 µL of this overnight culture was used to inoculate 3 mL of TYES broth. Deferiprone was added (Hider and Hoffbrand, 2018), and cultures were incubated at 28°C with rotation. Cultures were observed for turbidity after 24 hours. A range of 0 µM to 1 mM of deferiprone was tested in increments of 25 µM and the minimum concentrations that prevented growth of each strain are listed in **Table 3**. Experiments were performed twice in test tubes and once in a flask to confirm the inhibitory chelator concentrations. For some experiments ferric chloride (FeCl_3_) was added to the 3-ml TYES cultures containing deferiprone to determine the minimum amount of supplemental iron needed to restore turbid growth. A control tube (no *F. columnare* cells added) demonstrated that FeCl_3_ did not precipitate when added to medium containing deferiprone. A range of 0 µM to 200 µM of FeCl_3_ was tested in increments of 10 µM and the minimum concentrations that restored turbid growth for each strain are listed in **Table 3** Experiments were performed twice in test tubes and once in a flask to confirm the concentrations.

### Challenges of adult zebrafish

Adult zebrafish (*Danio rerio*) were challenged with *F. columnare* wild type and mutant strains as previously described (Conrad et al., 2022). In brief, bacterial strains were grown in TYES medium until OD_600_ reached 0.5. Cultures were serially diluted, plated on TYES agar, and incubated for 2 days at 28°C to determine the number of live cells per mL. To test the virulence of each strain, naïve adult Ekkwill zebrafish were immersed in a mixture of 0.5 mL *F. columnare* cells and 99.5 mL dechlorinated tap water for 30 minutes. No signs of disease were observed prior to challenge and no indications of *F. columnare* or columnaris disease were observed in the uninfected control tanks or in the maintenance tanks at any time. Control fish were exposed to a mixture of 0.5 mL *F. columnare ΔgldN* mutant and 99.5 mL of water for 30 minutes. After exposure, fish were moved to tanks containing 2 liters of fresh water at 28°C and observed for up to ten days for signs of infection. Each treatment was performed in triplicate tanks, with each tank containing five zebrafish. Mortalities were recorded daily. A minimum of 20% of fish that died were examined for bacteria phenotypic of *F. columnare* (yellow, rhizoid, tobramycin-resistant colonies) by swabbing the gills, fins and skin and streaking on TYES agar containing tobramycin (1 µg/mL), and incubating for 2 days at 30°C. Survivors from the adult zebrafish challenges were maintained for 28 days post infection. After 28 days, three survivors from each treatment (one from each tank) were exposed to Δ*gldN* mutant as a control while the rest were exposed to wild type *F. columnare* strain MS-FC-4. Fish were challenged using the method described above.

### Challenge of germ-free zebrafish larvae

Germ-free zebrafish (*Danio rerio*) larvae were challenged with wild-type, mutant, and complemented strains of *F. columnare* strain MS-FC-4 at 28°C as previously described (Stressmann et al., 2021). In brief, 10 to 12 germ-free larvae (6 days postfertilization) were exposed to 10^4^ CFU/mL *F. columnare* cells for 3 hours in 25-cm^3^ culture flasks with vented caps containing 20 mL of sterile mineral water. They were then transferred to individual wells of 24-well plates containing 2 mL sterile water per well. Larvae were fed every 48 h with 50 µL of germ-free *Tetrahymena thermophila* per well. Mortalities were counted daily and measured in days-post-infection (dpi) with 0 dpi corresponding to the infection day. All zebrafish larval experiments were stopped at 9 dpi, and zebrafish were euthanized with tricaine (MS-222) (Sigma- Aldrich; catalog no. E10521). Each experiment was repeated at least twice.

### Rainbow trout challenges

Commercially available certified disease-free rainbow trout (*Oncorhynchus mykiss*) eggs were acquired from Troutlodge Inc., Sumner, WA. Viable hatched trout were hand fed daily to satiation using a commercially available trout feed (Ziegler Inc., PA). Trout were maintained at the USDA-ARS National Center for Cool and Cold Water Aquaculture research facility in Kearneysville, WV in flow through water at a rate of 1 L/min, at 12.5°C, until the challenge weight of ~1.3 g was met. The fish in this facility are checked yearly for multiple diseases including columnaris disease, and except for fish in the challenge room, they are certified disease-free. No signs of disease were observed prior to challenge and no indications of *F. columnare* or columnaris disease were observed in the uninfected control tanks or in the maintenance tanks at any time. Fish were moved to challenge aquaria 1 week prior to immersion challenge to acclimate to the elevated water temperature of 16°C.

Wild-type (strain MS-FC-4), mutant and complemented strains were each used for immersion challenges. Frozen bacterial stocks were stored at -80°C in 75% TYES-2xMg broth and 25% glycerol. Bacterial cultures, for challenges, were grown as previously described with slight modifications (Evenhuis and LaFrentz, 2016). Briefly, 100 μl of each frozen stock was inoculated into 10 mL TYES-2xMg broth and incubated overnight at 30°C with shaking at 150 rpm. These starter cultures were used to inoculate 10 ml cultures (1:20 dilution) that were grown to OD_540_ of 0.4. Then, for each culture, ~6 ul was inoculated into 1 L TYES-2xMg broth in a 2.8 L Fernbach flask. These were incubated at 30°C with shaking at 150 rpm until OD_540_ of 0.5 to 0.6 was reached, at which point the cells were used for the challenge.

Challenges of fry were performed using triplicate 3 L tanks with restricted water flows (~200 mL/min) at 16°C. Each tank contained 40 fish of approximately 1.35 g each. Water flows were stopped for the immersion challenge and tanks were inoculated with bacterial cultures and incubated for 0.5 h after which water flows were resumed. Control tanks were inoculated with TYES-2xMg broth. Serial dilutions of water samples from each tank after inoculation were plated on TYES-2xMg agar to determine CFU/mL. The final challenge concentrations for each experiment are listed in the figures. Mortalities were removed and counted daily. The data for triplicate tanks of each strain were pooled and survivor fractions for each strain were calculated. Challenges continued for 21 days or until 3 days without recorded mortalities post-exposure. Approximately 16% of mortalities were randomly tested by homogenizing gill tissue and streaking on TYES-2xMg agar plates to determine if *F. columnare* was present. Confirmation of *F. columnare* was determined by morphological observation of yellow, rhizoid, adherent colonies and by amplifying 16S rRNA genes and confirming the genomovar by enzymatic digestion (HaeIII) and gel electrophoresis as previously described (Triyanto and Wakabayashi, 1999; Olivares-Fuster et al., 2007; LaFrentz et al., 2014). *F. columnare* was detected in all mortalities tested and all were genomovar I (and genetic group 1), as expected for strain MS-FC-4.

Challenges of rainbow trout alevin were performed as previously described (Evenhuis et al., 2021). In brief, 100 alevin were challenged 3 days post-hatch in 3-liter tanks. Total CFUs are given in the figures. Mortalities were removed daily and whole alevin were homogenized and streaked on TYES agar plates to determine the presence of *F. columnare*.

### Statistical analyses

For growth curve assays a one-way ANOVA with Tukey’s post-test was used to analyze differences between strains, unless otherwise noted. Error bars represent SEM (standard error of the mean). GraphPad Prism (version 9.1.2) was used to analyze infection challenges. A value of *p* < 0.05 was considered significant.

## Data availability statement

The original contributions presented in the study are included in the article/**Supplementary Material**. Further inquiries can be directed to the corresponding author.

## Ethics statement

Experiments with adult zebrafish followed protocols approved by the University of Wisconsin-Milwaukee Institutional Animal Care and Use Committee. Experiments with zebrafish larvae were performed according to European Union guidelines for handling laboratory animals and were approved by the Institut Pasteur institutional Animal Health and Care Committees under permit # dap200024. Rainbow trout challenges were performed as described in Protocol #176, which was approved by the NCCCWA Institutional Animal Care and Use Committee.

## Author contributions

RC and MM contributed to conception and design of the study and performed the bioinformatic analyses. MM, JE and J-MG obtained funding to support the research. RC constructed all mutants, performed growth experiments, and performed and analyzed all experiments involving adult zebrafish. D-PP, RS and J-MG performed and analyzed all experiments on zebrafish larvae. JE, RL and CB performed and analyzed all experiments involving rainbow trout. RC wrote the initial draft of the manuscript. All authors contributed to manuscript revision, read, and approved the submitted version.

## Funding

This work was funded in part by United States Department of Agriculture-ARS CRIS projects 8082-32000-006-00-D and 5090-31320-004-00D and by cooperative agreements 5090-31320-004-03S and 58-5090-1-022, and by grant NA18OAR4170097, project R/SFA-20 from the University of Wisconsin Sea Grant Institute under grants from the National Sea Grant College Program, National Oceanic and Atmospheric Administration, U.S. Department of Commerce, and the State of Wisconsin. J-MG, D-PP and RS were funded by the French government’s Investissement d’Avenir Program, Laboratoire d’Excellence “Integrative Biology of Emerging Infectious Diseases” (grant ANR-10-LABX-62-IBEID) and by an Institut Carnot Pasteur MS fellowship.

## Conflict of interest

The authors declare that the research was conducted in the absence of any commercial or financial relationships that could be construed as a potential conflict of interest.

## Publisher’s note

All claims expressed in this article are solely those of the authors and do not necessarily represent those of their affiliated organizations, or those of the funding agencies, the publisher, the editors and the reviewers. Any product that may be evaluated in this article, or claim that may be made by its manufacturer, is not guaranteed or endorsed by the publisher.
