# Supplementary material for: Flavobacterium columnare ferric iron uptake systems are required for virulence
Source: Front Cell Infect Microbiol. 2022 Oct 17;12:1029833. doi: 10.3389/fcimb.2022.1029833 (PMC9618737; doi:10.3389/fcimb.2022.1029833)
Supplement: Supplementary file 1 [file DataSheet_1.pdf]

Supplemental Figure and Tables for:

***Flavobacterium columnare* Ferric Iron Uptake Systems are Required for Virulence**

Authors: Rachel A. Conrad, Jason P. Evenhuis, Ryan S. Lipscomb, David Pérez-Pascual, Rebecca J. Stevick, Clayton Birkett, Jean-Marc Ghigo, Mark J. McBride

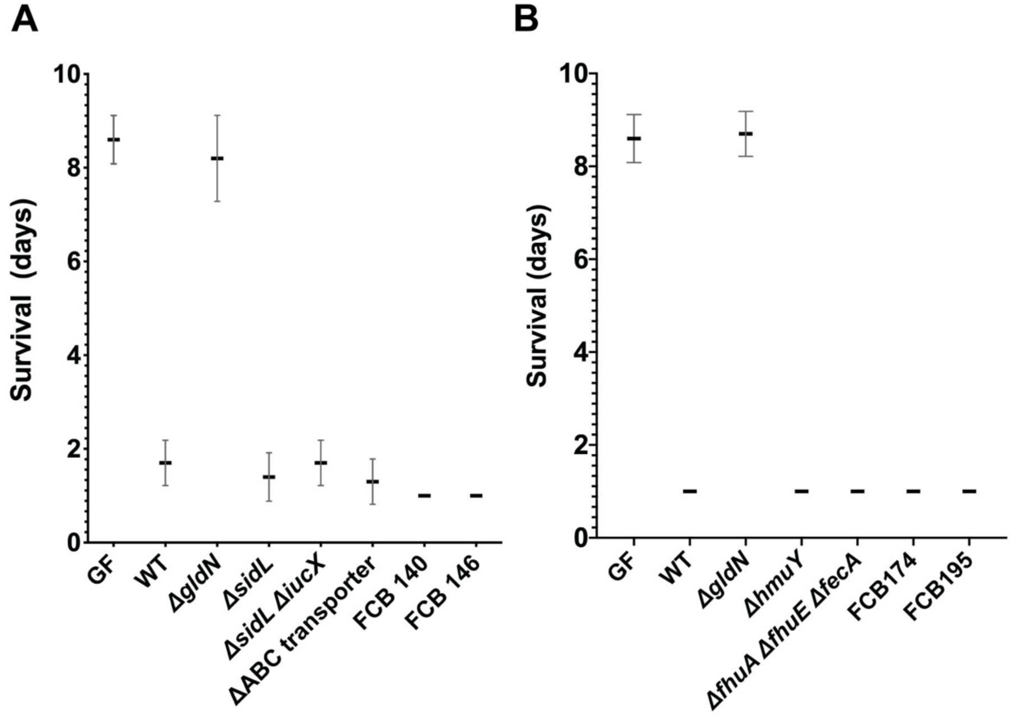

Figure S1. Virulence of *F. columnare* wild type and iron mutants toward germ-free (GF) zebrafish larvae. Fish were infected at 6 days postfertilization by immersion at 10^4 CFU/mL. Zero days post-infection (dpi) corresponds to the day of infection. Mean survival is represented by a thick horizontal bar with error bars for standard deviation. "GF" (germ-free) indicates noninfected larvae. **(A)** Length of survival for larvae exposed to WT, Δ*gldN*, Δ*sidL,* Δ*sidL* Δ*iucX*, ΔABC transporter, FCB140 (Δ*sidL* ΔABC transporter), and FCB146 (Δ*sidL* ΔABC transporter Δ*fhuA*). **(B)** Length of survival for larvae exposed to WT, Δ*gldN,* Δ*hmuY,* Δ*fhuA* Δ*fhuE* Δ*fecA,* FCB174 (Δ*sidL* ΔABC transporter Δ*fhuA* Δ*iucX)*, and FCB195 (Δ*sidL* ΔABC transporter Δ*fhuA* Δ*iucX* Δ*fhuE*). The number of days of survival for fish challenged with any of the iron gene deletion mutant strains were not significantly different from those of the wild type. The survival for fish challenged with the Δ*gldN* mutant was not significantly different from the non-infected GF control.

Supplemental Tables:

Table S1. Genes encoding proteins predicted to function in *F. columnare* strain MS-FC-4 ferrous iron acquisition

Locus Tag	Protein Name	NCBI Definition	Conserved Domains[a]	Predicted Function[b]	Protein Localization[c]
C6N29_06890	FeoB	Ferrous iron transporter B	FeoB (COG0370, TIGR00231, TIGR00437, pfam0221, pfam07664)	Ferrous iron transport system protein	Inner Membrane
C6N29_06895	FeoA	Ferrous iron transport protein A	FeoA (pfam04023, COG1918)	Ferrous iron transport system protein	Cytoplasmic

[a] Conserved domains as assigned by NCBI and by the Joint Genome Institute Integrated Microbial Genomes & Microbiomes (IMG/M version 6.0 [https://img.jgi.doe.gov/m]) (Chen et al., 2021). TIGRFAM, pfam, smart, cl, cd, or COG numbers are indicated.

[b] Function predicted based on conserved domains and gene organization.

[c] Location of each protein was predicted using psortb 3.0 (Yu et al., 2010).

Table S2. Plasmids used in this study.

Plasmid	Description[a]	Source or reference
pCP23	*E. coli-F. columnare* shuttle plasmid; Ap^r $(Tc)^r$	(Agarwal et al., 1997)
pMS75	Suicide vector carrying *sacB*; Ap^r (Tc^r)	(Li et al., 2015)
pRC18	2.1 kbp region downstream of *hmuY* amplified with primers 2173 and 2174 and cloned into BamHI and SalI sites of pMS75; Ap^r (Tc^r)	This study
pRC19	2.1 kbp region upstream of *hmuY* amplified with primers 2171 and 2172 and cloned into KpnI and BamHI sites of RC18; Ap^r (Tc^r)	This study
pRC36	2.2 kbp region downstream of *fhuA* amplified with primers 2388 and 2389 and cloned into BamHI and SalI sites of pMS75; Ap^r (Tc^r)	This study
pRC37	2.1 kbp region upstream of *fur* amplified using primers 2392 and 2393 and cloned into BamHI and SalI sites of pMS75; Ap^r (Tc^r)	This study
pRC38	2.2 kbp region upstream of *fhuA* amplified with primers 2386 and 2387 and cloned into KpnI and BamHI sites of pRC36; Ap^r (Tc^r)	This study
pRC41	2.2 kbp region downstream of *fur* amplified using primers 2390 and 2391 and cloned into KpnI and BamHI sites of pRC37; Ap^r (Tc^r)	This study
pRC42	2.2 kbp region downstream of the ABC transporter genes amplified with primers 2384 and 2385A and cloned into BamHI and SphI sites of pMS75; Ap^r (Tc^r)	This study
pRC43	2.2 kbp region upstream of the ABC transporter genes amplified with primers 2382A and 2383A and cloned into KpnI and BamHI sites of RC42; Ap^r (Tc^r)	This study
pRC44	2.2 kbp region downstream of the siderophore biosynthesis genes amplified using primers 2465 and 2466 and cloned into KpnI and BamHI sites of pMS75; Ap^r (Tc^r)	(Conrad et al., 2022)
pRC45	2.1 kbp region upstream of the siderophore biosynthesis genes amplified using primers 2467 and 2468 and cloned into BamHI and SalI sites of RC44; Ap^r (Tc^r)	(Conrad et al., 2022)
pRC47	3.1 kbp fragment containing the ABC transporter genes amplified using primers 2540 and 2541 and cloned into KpnI and PstI sites of pCP23; Ap^r (Tc^r)	This study
pRC51	2.3 kbp region upstream of *fhuE* amplified using primers 2550 and 2551 and cloned into BamHI and SalI sites of pMS75; Ap^r (Tc^r)	This study
pRC52	2.0 kbp region upstream of *fecA* amplified using primers 2554 and 2555 and cloned into KpnI and BamHI sites of pMS75; Ap^r (Tc^r)	This study

pRC53	2.3 kbp region downstream of *fhuE* amplified using primers 2548 and 2549A and cloned into KpnI and BamHI sites of pRC51; Ap^r (Tc^r)	This study
pRC54	2.6 kbp fragment containing *fhuE* amplified using primers 2552 and 2553A and cloned into KpnI and PstI site of pCP23; Ap^r (Tc^r)	This study
pRC55	2.6 kbp fragment containing *fecA* amplified using primers 2558A and 2559 and cloned into KpnI and PstI sites of pCP23; Ap^r (Tc^r)	This study
pRC56	1.8 kbp fragment containing *iucX* amplified using primers 2564 and 2565 and cloned into KpnI and PstI sites of pCP23; Ap^r (Tc^r)	(Conrad et al., 2022)
pRC57	2.2 kbp region downstream of *iucX* amplified using primers 2560 and 2561 and cloned into BamHI and SalI sites of pMS75; Ap^r (Tc^r)	(Conrad et al., 2022)
pRC58	2.6 kbp region upstream of *iucX* amplified using primers 2562 and 2563 and cloned into SalI and SphI sites of pRC57; Ap^r (Tc^r)	(Conrad et al., 2022)
pRC59	2.3 kbp region downstream of *fecA* amplified using primers 2556B and 2557A and cloned into BamHI and SalI sites of pRC52; Ap^r (Tc^r)	This study
pRC60	2.1 kbp region downstream of *fhuE* amplified using primers 2549A and 2581 and cloned into XmaI and BamHI sites of pRC51. This plasmid was used to construct FCB195; Ap^r (Tc^r)	This study
pRC68	6.9 kbp region containing upstream, ABC transporter genes, and downstream amplified using primer 2382A and 2385A and cloned into KpnI and SphI cut sites of pMS75; Ap^r (Tc^r)	This study
pRC69	6.3 kbp region containing upstream, *iucX* (C6N29_04155), and downstream amplified using primers 2560 and 2563 and cloned into BamHI and SphI cut sites of pMS75; Ap^r (Tc^r)	(Conrad et al., 2022)
pRC72	6.7 kbp region containing upstream, *fhuE* (C6N29_04165), and downstream amplified using primers 2606A and 2607 and cloned into BamHI and SalI cut sites of pMS75. This plasmid was used to complement FCB195; Ap^r (Tc^r)	This study
pRC73	6.5 kbp region containing upstream, *fhuE* (C6N29_04165), and downstream amplified using primers 2605A and 2606A and cloned into BamHI and SalI cut sites of pMS75. This plasmid was used to complement FCB160; Ap^r (Tc^r)	This study

[a]Antibiotic resistance phenotypes: ampicillin, Ap^r; tetracycline, (Tc^r). Unless indicated otherwise, the antibiotic resistance phenotypes are those expressed in *E. coli*. The antibiotic resistance phenotypes given in parentheses are those expressed in *F. columnare* but not in *E. coli*.

Table S3. Primers used in this study

Primer	Sequence (5' to 3')[a]	Plasmid constructed using this primer
2171	GCTAGGGTACCGGTTCTTGTAACTTAAATATCAGTGGAAA	pRC19
2172	GCTAGGGATCCGAAATAGATTTACCAGAAAATATGAATAAAGGT	pRC19
2173	GCTAGGGATCCGTAAACAGACCAATTGAAAATCATCAA	pRC18
2174	GCTAGGTCGACGCTAGTCTAAAACGAGTACAAGAG	pRC18
2382A	GCTAGGGTACCAACTTGCCCTATCTGCAAAG	pRC43, pRC68
2383A	GCTAGGGATCCAGCAGTGGCATCTGTTAGAAT	pRC43
2384	GCTAGGGATCCGAGGATGAAGACATTCTATTTGATAAC	pRC42
2385A	GCTAGGCATGCACTTGCCTTTCGAATGCTC	pRC42, pRC68
2386	GCTAGGGTACCGGATTGCTAATTACACCGCA	pRC38
2387	GCTAGGGATCCAACCTATCTTGACCGAAACT	pRC38
2388	GCTAGGGATCCTACAGTATTAATCCAATACCGCCA	pRC36
2389	GCTAGGTCGACTGAGCCAATCGCCAAATAGA	pRC36
2390	GCTAGGGTACCGCCTACAAGAACAACTCGATC	pRC41
2391	GCTAGGGATCCAATCATTCATTATATTTCTACGCTACT	pRC41
2392	GCTAGGGATCCTGTAACTTGTGTCATAACGTCACA	pRC37
2393	GCTAGGTCGACTCGCAATCATTACAATGGCT	pRC37
2465	GCTAGGGTACCACCGCAGAGTTTTGGTTGAA	pRC44
2466	GCTAGGGATCCCTTGGTTTTTGGGTTTTTCAG	pRC44
2467	GCTAGGGATCCAGCAAATTTGTTTGCAGTCCC	pRC45
2468	GCTAGGTCGACTGCATCGCCGTGTGTACTAT	pRC45
2540	GCTAGGGTACCTACAATTGATTAATACTTCTTTTTAAGGCA	pRC47
2541	GCTAGCTGCAGCTTTGGTGGTTAACGGATCAA	pRC47
2548	GCTAGGGTACCTAGCGGGCCTTGTTTATTTG	pRC53
2549A	GCTAGGGATCC GAAAAAGGATTGTGGGGCTTT	pRC53, pRC60
2550	GCTAGGGATCCGTCTTGACAATAGGAAATCATTGC	pRC51
2551	GCTAGGTCGACGAGTGTTGGCAGGTGACTT	pRC51
2552	GCTAGGGTACCTTATCAAAGCAGGCGACC	pRC54
2553A	GCTAGCTGCAGAGCTGTGGATGTTGCGTA	pRC54
2554	GCTAGGGTACCGCCCTTTTAATGTTTTATACAGGA	pRC52
2555	GCTAGGGATCCACCAATTATGGTATTTTGCGA	pRC52

2556B	GCTAGGGATCCGTGTTGGACGTATCTGCTTCT	pRC59
2557A	GCTCCGTCGACCTTTCACCTCCCAAAGTCTGA	pRC59
2558A	GCTAGGGTACCTGGCTCTTAATGGTCGATTGA	pRC55
2559	GCTAGCTGCAGCGAAGTCTGATGCGTAATCTG	pRC55
2560	GCTAGGGATCCAACGATTTCGTTGCTTCAGG	pRC57, pRC69
2561	GCTAGGTCGACATGCGGGTAGAAGACAAAACC	pRC57
2562	GCTAGGTCGACGATGTTTGTTCTTAAATCTTTCCA	pRC58
2563	GCTAGGCATGCCACAGCAACCTTATCCGT	pRC58, pRC69
2564	GCTAGGGTACCATGCAAGTAAAAGCGACATCC	pRC56
2565	GCTAGCTGCAGAGTGCCATGTATTCACCCAAA	pRC56
2581	GCTAGCCCGGG ATCACTCCCGAGAACAATGC	pRC60
2605A	GCTAGGGATCCAACATAAAGGAGAGTAGCGG	pRC73
2606A	GCTAGGTCGACTGGAAGAACCAACCATTAGC	pRC72, pRC73
2607	GCTAGGGATCCCGCCCATTGTGAGTGTTGTC	pRC72

[a] Underlined sequences indicate added restriction enzyme sites.

References:

Agarwal, S., Hunnicutt, D.W., and McBride, M.J. (1997). Cloning and characterization of the *Flavobacterium johnsoniae* (*Cytophaga johnsonae*) gliding motility gene, *gldA*. *Proc. Natl. Acad. Sci. USA* 94**,** 12139-12144.

Chen, I.M.A., Chu, K., Palaniappan, K., Ratner, A., Huang, J.H., Huntemann, M., et al. (2021). The IMG/M data management and analysis system v.6.0: new tools and advanced capabilities. *Nucleic Acids Research* 49(D1)**,** D751-D763. doi: 10.1093/nar/gkaa939.

Conrad, R.A., Evenhuis, J.P., Lipscomb, R., Birkett, C., and McBride, M.J. (2022). Siderophores produced by the fish pathogen *Flavobacterium columnare* strain MS-FC-4 are not essential for its virulence *Appl. Environ. Microbiol.* 88**,** e0094822.

Li, N., Qin, T., Zhang, X.L., Huang, B., Liu, Z.X., Xie, H.X., et al. (2015). Gene deletion strategy to examine the involvement of the two chondroitin lyases in *Flavobacterium columnare* virulence. *Appl Environ Microbiol* 81(21)**,** 7394-7402. doi: 10.1128/AEM.01586-15.

Yu, N.Y., Wagner, J.R., Laird, M.R., Melli, G., Rey, S., Lo, R., et al. (2010). PSORTb 3.0: Improved protein subcellular localization prediction with refined localization subcategories and predictive capabilities for all prokaryotes. *Bioinformatics* 26**,** 1608-1615.
